# Anaerobic Membrane Bioreactors for Municipal Wastewater Treatment: A Literature Review

**DOI:** 10.3390/membranes11120967

**Published:** 2021-12-08

**Authors:** Yerkanat N. Kanafin, Dinara Kanafina, Simos Malamis, Evina Katsou, Vassilis J. Inglezakis, Stavros G. Poulopoulos, Elizabeth Arkhangelsky

**Affiliations:** 1 Environmental Science & Technology Group (ESTg), The Environment & Resource Efficiency Cluster (EREC), Chemical and Materials Engineering Department, School of Engineering and Digital Sciences, Nazarbayev University, Nur-Sultan 010000, Kazakhstan; yerkanat.kanafin@nu.edu.kz (Y.N.K.); dinara.kanafina@nu.edu.kz (D.K.); stavros.poulopoulos@nu.edu.kz (S.G.P.); 2 Department of Water Resources and Environmental Engineering, School of Civil Engineering, National Technical University of Athens, 15780 Athens, Greece; malamis.simos@gmail.com; 3 Department of Civil and Environmental Engineering, College of Engineering, Design and Physical Sciences, Brunel University London, Uxbridge UB8 3PH, UK; evina.katsou@brunel.ac.uk; 4 Chemical and Process Engineering Department, Faculty of Engineering, University of Strathclyde, Glasgow G1 1XJ, UK; vasileios.inglezakis@strath.ac.uk; 5 Environmental Science & Technology Group (ESTg), The Environment & Resource Efficiency Cluster (EREC), Civil and Environmental Engineering Department, School of Engineering and Digital Sciences, Nazarbayev University, Nur-Sultan 010000, Kazakhstan

**Keywords:** anaerobic digestion, membrane bioreactor, wastewater treatment

## Abstract

Currently, there is growing scientific interest in the development of more economic, efficient and environmentally friendly municipal wastewater treatment technologies. Laboratory and pilot-scale surveys have revealed that the anaerobic membrane bioreactor (AnMBR) is a promising alternative for municipal wastewater treatment. Anaerobic membrane bioreactor technology combines the advantages of anaerobic processes and membrane technology. Membranes retain colloidal and suspended solids and provide complete solid–liquid separation. The slow-growing anaerobic microorganisms in the bioreactor degrade the soluble organic matter, producing biogas. The low amount of produced sludge and the production of biogas makes AnMBRs favorable over conventional biological treatment technologies. However, the AnMBR is not yet fully mature and challenging issues remain. This work focuses on fundamental aspects of AnMBRs in the treatment of municipal wastewater. The important parameters for AnMBR operation, such as pH, temperature, alkalinity, volatile fatty acids, organic loading rate, hydraulic retention time and solids retention time, are discussed. Moreover, through a comprehensive literature survey of recent applications from 2009 to 2021, the current state of AnMBR technology is assessed and its limitations are highlighted. Finally, the need for further laboratory, pilot- and full-scale research is addressed.

## 1. Introduction

Conventional activated sludge wastewater treatment plants (WWTPs), in many cases, do not reach the strict quality limits for effluent reuse, while the energy potential of the chemical bonds of the organic substances and the thermal energy of the sewage remains unused. The upgrading of existing WWTPs is needed but also the shift to new innovative technologies that can meet modern requirements.

Due to the strengthening of discharge standards worldwide, membrane bioreactors (MBRs) are being widely applied. Essentially, MBR is an integration of the conventional activated sludge process with membrane technology [1]. The anaerobic membrane bioreactor (AnMBR) is one of the configurational types of MBRs. It is advantageous over aerobic MBR due to the higher quality of the effluent and lesser amounts of sludge produced [2].

The anaerobic treatment of municipal wastewater is based on the biological process in which certain microorganisms, in the absence of oxygen, decompose complex organic compounds into simpler ones and eventually convert them to methane (CH_4_) and carbon dioxide (CO_2_) [3]. The process decelerates under cold conditions and needs mesophilic or thermophilic conditions (about 35 °C or higher) for stable operation [4,5,6]. Moreover, it is not possible to generate enough biogas due to the low organic content of municipal wastewater [7]. The limitations reported can only be addressed by the selection of suitable anaerobic systems designed to make anaerobic treatment efficient in both the management of large volumes of urban wastewater and at low ambient temperatures [7]. The need for high-quality effluent and the need to save energy are some of the reasons that have led to the development of new methods of urban wastewater treatment. AnMBR technology is one of the efficient technologies in the field of urban wastewater management based on anaerobic treatment.

The development of bioreactors, whose function is based on the formation of biofilms that act as a barrier to the passage of biomass, has been a milestone in anaerobic wastewater treatment technology. The membrane technology works in the same way, separating hydraulic retention time from solids retention time, allowing the application of the AnMBR technology to urban wastewater treatment.

The majority of previously published reviews on AnMBRs in the field have focused on issues of membrane filtration, such as membrane fouling, the configurational type of modules and on energy consumption. This paper aims at exploring the fundamental aspects of AnMBRs for municipal wastewater treatment. Special attention is given to describing the anaerobic processes and to evaluate the impact of different parameters, such as pH, temperature, alkalinity, volatile fatty acids, organic loading rate, hydraulic retention time and solids retention time. The extensive review table, which covers the papers from 2009 to 2021, can serve as a dataset for exploring the past works and for further analysis of the researchers in the interdisciplinary field.

## 2. Anaerobic Degradation and AnMBR Configurations and Bioreactors

### 2.1. Anaerobic Degradation

**Figure 1:**
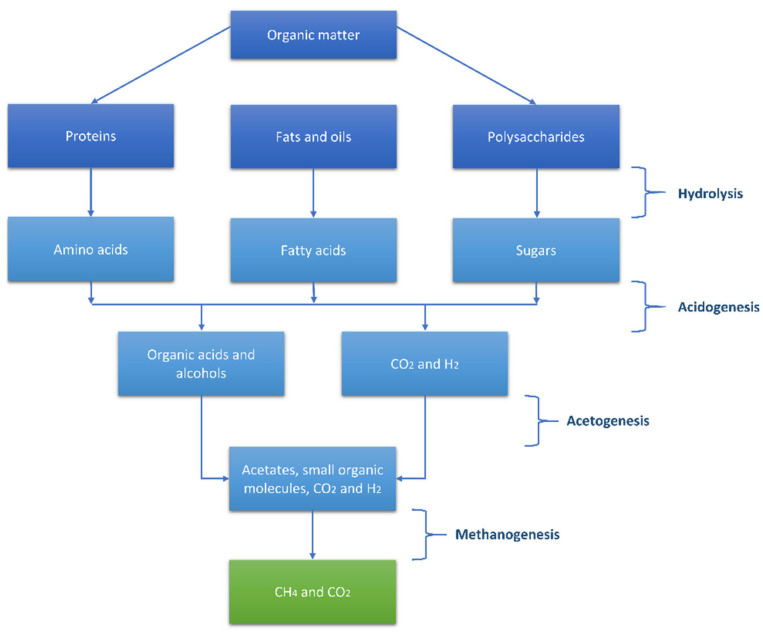
Anaerobic treatment stages.

The anaerobic degradation of organic substances is carried out by anaerobic microorganisms (Figure 1). A crucial process that enables the metabolism of organic compounds by anaerobic microorganisms is hydrolysis, that is, their degradation into compounds of lower molecular weight so that they can enter the cells of the microbes. This step is achieved by the microorganisms through the release of specific proteins, the enzymes (enzymatic hydrolysis) outside of their cells. Enzymes catalyze hydrolysis reactions, in which organic polymers, such as lipids, proteins and polysaccharides, are broken down into simpler organic compounds. Lipids are broken down into fatty acids and glycerol, proteins into amino acids and polysaccharides into monosaccharides (glucose, fructose, galactose). Organic compounds are then available to enter the cytoplasm of microorganisms and begin the process of metabolism which is completed in three stages:

(i) Acidogenesis, during which the products of hydrolysis (sugars, fatty acids, amino acids) are taken up by acidogenic bacteria and are metabolized to short chain fatty acids (lactic acid, propionic acid, butyric acid), ethanol, hydrogen and carbon dioxide;
(ii) Acetogenesis, where the intermediate compounds of acidogenesis are converted through the function of anaerobic bacteria into acetic acid, hydrogen and carbon dioxide.
(iii) Methanogenesis where methanogenic bacteria produce methane and carbon dioxide and therefore biogas.

A characteristic of anaerobic treatment is the slow rate of growth of anaerobic bacteria.

The process of anaerobic degradation is carried out under specific conditions of temperature, pH and alkalinity, and its course depends on factors such as the composition of the organic matter, the concentration of nutrients and the presence of toxic substances that can inhibit the rate of degradation. In anaerobic treatment, the rate-limiting step is the hydrolysis of particulates to soluble substrates [7]. The hydrolysis of particulates relies on the aforementioned process parameters and the biomass, which is the source of hydrolytic enzymes. The rate of hydrolysis is affected by the presence of fats and suspended solids in the organic material. Moreover, the hydrolysis products can also inhibit the activity of hydrolytic enzymes [8].

In addition, in an anaerobic membrane bioreactor (AnMBR) system treating municipal wastewater, the parameters that determine the course and performance of the biological process are the hydraulic retention time (HRT) and the sludge retention time (SRT) and organic loading rate [3]. The efficiency is mainly related to the amount of biogas produced. AnMBR technology is considered as a low-energy footprint technology.

The anaerobic treatment has many limitations for efficient application in municipal waste treatment. The slow rate of proliferation of anaerobic bacteria, especially in cold environmental conditions, requires long hydraulic residence times of the effluent in the anaerobic reactor in order to achieve the biodegradation of organic matter. Consequently, large-volume bioreactors are needed to manage large volumes of urban wastewater [4,5,6].

In order to facilitate, or better to accelerate, the biological activity of the bacteria, and thus the biodegradation of the organic wastewater components, the bioreactor temperature should be increased to mesophilic conditions (about 35 °C). However, increasing the temperature requires energy consumption and therefore significantly increases the cost of the process [4,5,6].

Another limitation is the low organic load of sewage. When the ambient temperature is around 15 °C, for the anaerobic biological activity to be effective, the chemical oxygen demand (COD) levels of the incoming effluent should be higher than 4–5 g/L [4,5,6]. This is a barrier to the application of the method to low-organic load urban wastewater which usually has a COD less than 1000 mg/L) [7].

In fact, initially, the applications of anaerobic sewage treatment involved industrial wastewater with biodegradable COD >4000 mg/L in tropical areas with an ambient temperature of not less than 20–25 °C. Subsequently, anaerobic treatment was applied to urban sewage only in developing areas with high ambient temperatures, such as Brazil, Colombia, Mexico, Egypt and India [7].

### 2.2. AnMBR Configurations

**Figure 2:**
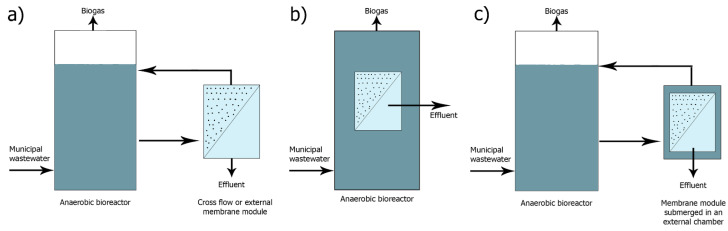
Simplified illustration of (**a**) an external/pressurized AnMBR; (**b**) a submerged AnMBR; and (**c**) externally submerged AnMBR.

The first commercial anaerobic membrane system was developed in 1980 by Dorr–Oliver to process high-organic load wastewater and in particular dairy wastewater. However, it has only been applied on a pilot scale, perhaps due to the high cost of membranes [1]. Since then, AnMBR systems have been studied for the treatment of industrial as well as municipal wastewater [7]. Three main layouts of the AnMBR system are shown in Figure 2.

The first variation is an anaerobic reactor with an external membrane unit (crossflow AnMBR or external AnMBR) (Figure 2a). In the outer configuration, the membrane unit is separate from the reactor. The mixed liquor is fed under pressure from the bioreactor to the membrane unit. The membranes, operating at a tangential flow and under pressure, produce the permeate (treated effluent). The retentate which is concentrated on one side of the membrane is recirculated to the anaerobic bioreactor [9].

The second type is submerged AnMBR, where the membrane unit is directly immersed in the anaerobic bioreactor (Figure 2b). The membranes come into direct contact with the dissolved anaerobic biomass contained therein. Inside the membranes, low negative pressure is applied and this pressure difference between the mixed liquor on one side of the membrane and low negative pressure on the other side of the membrane is the driving force for the filtration process [9].

Finally, an external tank anaerobic reactor system with an immersed membrane unit (externally submerged AnMBR) is the third configuration (Figure 2c). The membrane system may be in an external assembly separate from the main bioreactor, immersed in a tank filled with biomass and operating at low negative pressure. In such an external submerged assembly, the biomass is pumped from the bioreactor to the external assembly, while the excess amount of mixed liquor is recycled to the bioreactor [9].

The outer membrane assemblies have the significant advantage of easy replacement or cleaning of the membranes without disturbing the anaerobic reactor operating conditions [9]. In the external AnMBR, the pumps are used to recycle the retentate. This recirculation contributes to a high shear rate that can break the cells and flocs and prevent the membrane fouling [10].

In recent years, however, submerged systems have become prevalent for economic reasons. The main advantage of immersed systems compared to external systems is that they have lower energy requirements, as filtration takes place at lower pressures [11]. Furthermore, external systems require more space and the tanks that need to be built are more costly.

### 2.3. Bioreactors

**Figure 3:**
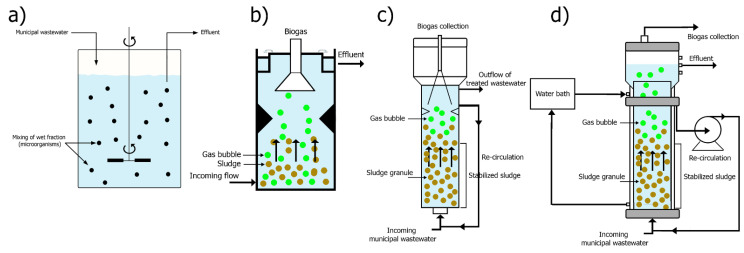
Schematic representation of (**a**) the CSTR bioreactor; (**b**) the upstream flow anaerobic bed reactor; (**c**) the EGSB bioreactor and (**d**) the fluidized bed reactor.

The anaerobic treatment of municipal wastewater is usually carried out in continuously stirred tank reactors (CSTRs), upflow anaerobic sludge blanket (UASB) reactors, expanded granular sludge bed reactors (EGSBs) and fluidized bed reactors (Figure 3). Hybrid bioreactor systems or arrays can also be used [12,13,14,15,16]. The CSTR bioreactor is being further investigated in AnMBR systems [12]. Full mixing is achieved in circular or square tanks with mechanical stirrers or liquid or biogas re-circulation (Figure 3a). Its dimensions are determined by the time required for the wastewater stream (feed stream) to remain in the bioreactor [17].

The UASB bioreactor was invented in the 1970s by Lettinga in the Netherlands (Figure 3b) [7]. The biggest success of the UASB is that it enables the maintenance of a high biomass concentration due to the formation of a dense sludge layer at the bottom of the reactor. An appropriate arrangement (gas–liquid–solid separator) is made to collect the biogas produced from the upper part of the reactor. The formation of a stabilized sludge layer acts as a barrier that internally retains the solids of the incoming wastewater stream. Therefore, it allows the hydraulic retention time to be separated from the sludge retention time. The treatment can be efficient and carried out at high organic load with a significant reduction in reactor volume [12]. The success of the separation of the hydraulic retention time of the wastewater from the solids retention time provided the opportunity to develop various methods for internal retention or external separation of biomass from the liquid fraction. Membrane technology is one of these methods [12].

The EGSB bioreactor is a variant of the UASB bioreactor (Figure 3c). What distinguishes it is the higher upstream flow rate of the feed stream passing through the bottom sludge layer. The flow rate allows the granular sludge to expand, improving its mixing with the liquid fraction. Separation of dissolved constituents from the sludge layer is also achieved. The increased flow speed requires high reactors.

In the fluidized bed reactor, the feed stream flows from the bottom upwards through the bed and keeps the bed fluidized, constantly changing its volume (Figure 3d). This movement allows the fluid and particles of the feed stream to mix well to facilitate the biological processes. The feed stream then leaves the reactor, and part of it returns to the bottom of the outer recirculation.

The membrane unit is immersed in the bioreactors or placed in an external tank. The latter configuration allows a large amount of biomass to be kept in the bioreactor, so that the amount of biomass that comes into contact with the membranes decreases. This limits membrane fouling [1]. However, the development of biomass on the membrane surface, colloids, soluble microbial products and extracellular polymeric constituents (such as hydrocarbons, proteins, lipids and nucleic acids) contribute significantly to membrane fouling. Consequently, the design of bioreactors that limit membrane–biomass contact cannot guarantee a reduction in membrane fouling [9].

## 3. The Impact of Operating Conditions on the Process

### 3.1. Temperature

Temperature is a determining factor in any biological process. In general, it affects the growth rate of the microbial population, the hydrolysis of organic components and the solubility of components, such as CH_4_ and CO_2_.

**Figure 4:**
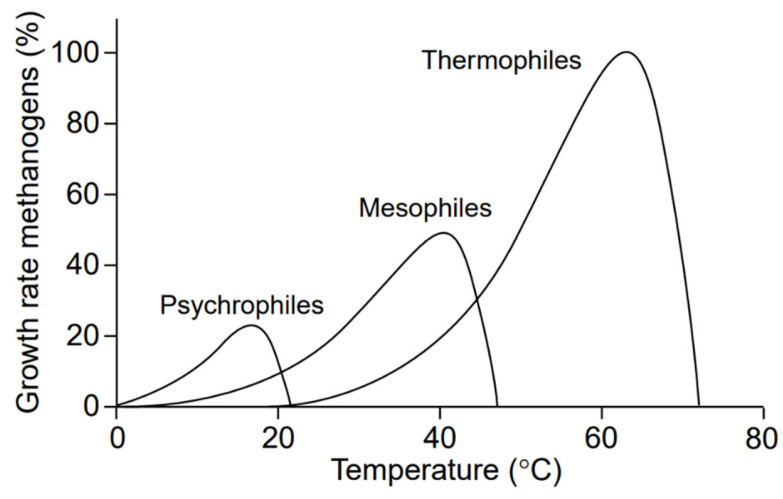
Growth rate of methanogenic bacteria in relation to temperature. Reprinted from ref. [18], copyright (2001), with permission from Elsevier.

In theory, increasing the temperature at which anaerobic wastewater is treated increases the metabolism of microorganisms, facilitates hydrolysis and accelerates the methanogenesis step. This contributes to high system efficiency in the collection of biogas produced. Of course, there is a limit to the increase in temperature, beyond which anaerobic degradation becomes difficult and the AnMBR system is destabilized (Figure 4).

Municipal wastewater has a temperature range that belongs to the cold-water area (<20 °C), particularly in Central and Northern Europe. At this temperature, the process of hydrolysis and the dissolution of complex organic constituents into soluble forms that can feed microorganisms is a limiting factor. In addition, the development of slow-growing methanogenic microorganisms is not favored [18].

Anaerobic municipal wastewater degradation can therefore be carried out satisfactorily as far as biogas production is concerned in two temperature zones: mesophilic (28–45 °C) and thermophilic (>60 °C) [19]. Anaerobic treatment at lower temperatures can also occur but with much lower efficiency.

Concerning the removal of organic material as dissolved COD, Skouteris et al. reported that when the temperature decreased from 25 °C to 15 °C the removal of soluble COD decreased from 95% to 85% [20].

In addition to the metabolic rates of microorganisms, temperature also affects such parameters as the solubility of biogas [12]. The methane produced is more soluble at lower temperatures (<20 °C) and this results in higher losses as a soluble component of the filtrate [21]. At 20 °C, the solubility of methane is 30% higher than its solubility at 35 °C [21].

Temperature fluctuations have an important effect on anaerobic degradation. Mesophilic bacteria are resistant to fluctuations of ±3 °C, but thermophiles are more sensitive and require longer adaptation to new conditions [22].

### 3.2. pH

The methanogenic bacteria grow in an optimum pH range of 6.5–8.2. However, the steps of hydrolysis and acidogenesis require a pH of 5.5–6.5 [23]. In general, pH is an important parameter that should be controlled in AnMBR systems, as higher or lower values can hinder the process. pH affects the process directly, altering the protein structure of the enzymes, but also indirectly, affecting the toxicity of the various components.

During the process of anaerobic degradation of the organic constituents, volatile fatty acids are formed which reduce the pH value. A large pH drop can be detrimental to the subsequent methanogenesis step. However, during protein degradation, ammonium (NH_4_^+^) cations are abundantly produced and CO_2_ solubilization produces bicarbonate ions (HCO_3_^−^) which resist pH reduction due to buffer capacity and restore its value (Equation (1)) [24].

(1) $${\mathrm{NH}}_{3}+H_{2}O+{\mathrm{CO}}_{2}\to {\mathrm{NH}}_{4}^{+}+{\mathrm{HCO}}_{3}^{-}$$

The coexistence of ammonium and bicarbonate ions is critical and gives the system a regulatory capacity so that the pH can withstand changes and maintain a constant value [23,25].

Temperature also has a significant influence on the pH value of the anaerobic system. When the temperature is below 20 °C, the gases produced, such as methane and carbon dioxide, become more soluble, leading to an increase in pH [26]. The temperature, the buffering capacity of a solution and the concentration of volatile acids are three interrelated factors that play an important role in the proper operation of the anaerobic system.

Finding the optimum pH value can promote methane production. Based on previous works, maximum biogas production can be achieved at pH 7.0 and is equal to 0.4535 LCH_4_/gVS and decreases when pH reaches values of 6.0 (0.1889 LCH_4_/gVS) and 8.0 (0.2659 LCH_4_/gVS) [23].

As the value of pH increases, so does the solubility of CO_2_, which is converted to bicarbonate (HCO_3_^−^) and carbonate (CO_3_^−2^) ions, as well as hydroxyl (OH^−^) ions. At a pH value up to 4.3, there are no buffering ions in the solution; as the pH value increases, it is noted that the solution acquires a buffering capacity [27].

The design of AnMBR technology is currently being studied in separate two-stage phases to achieve optimum methane production. In this way it is possible to independently adjust the pH for the phase of acidogenesis in the first reactor and for methanogenesis in the second reactor. Indeed, while the optimum pH value for hydrolysis and acidogenesis is between 5.5 and 6.5, for methanogenesis the optimum is about 7 [24]. For example, Wijekoon et al. used a hydrolytic reactor at pH 5.5 and a methanogenic reactor at pH 7.2 and achieved 71% COD removal and 96% biological oxygen demand (BOD) removal [28].

### 3.3. Volatile Fatty Acids (VFAs)

Volatile fatty acids (VFAs) are produced during the hydrolysis phase. These are organic acids with a small number of carbon atoms in their chain and are the substrate to be used by methanogenic bacteria [24]. Although acetic acid is present at higher concentrations than other fatty acids, propionic and butyric acid are more likely to affect methanogenic bacteria. It is crucial that their concentration in the anaerobic reactor is maintained at a specific value range, otherwise the system loses its stability. VFAs are capable of intercepting anaerobic processing when formed at high concentrations, causing a decrease in the pH value [24]. Their effect is greater in AnMBR systems operating at low pH values [3]. The increased concentration and accumulation of VFAs is mainly due to overloading of the system but does not always lead to a decrease in pH due to the alkalinity of the wastewater that is fed [24]. However, the excessive accumulation of VFAs in an AnMBR can result in a decrease of the pH in the reactor, leading to an inhibition of the methanogenic bacteria.

### 3.4. Alkalinity

Alkalinity is the ability of a solution to neutralize acids and maintain the pH of the solution at a particular value. In wastewater, alkalinity is mainly due to carbonates (CO_3_^2−^), bicarbonates (HCO_3_^−^) and hydroxyl ions (OH^−^) and is usually in the range of 210–350 mgCaCO_3_/L. When the pH of the wastewater is higher than 6.6, the alkalinity should not be lower than 236 mgCaCO_3_/L for the anaerobic treatment to be achieved efficiently [26]. In cold conditions, during the anaerobic treatment, the pH value decreases due to the increased solubility of the gaseous products, such as CO_2_. A fall in pH value can threaten the smooth operation of the process and therefore high alkalinity is desirable. Alkalinity is usually regulated by the addition of sodium bicarbonate (NaHCO_3_) [26]. Calcium hydroxide (Ca(OH)_2_), calcium oxide (CaO) or sodium carbonate (Na_2_CO_3_) may also be added to regulate alkalinity [24].

One way to assess the stability of the anaerobic system is to calculate the intermediate alkalinity/partial alkalinity (IA/PA) ratio. The methodology uses the pH values 5.75 and 4.3 as reference points. Between the values of 5.75 and 4.3, alkalinity approximates the concentration of volatile fatty acids formed in the anaerobic reactor. Thus, the ratio numerator, intermediate alkalinity (IA), expresses the alkalinity of volatile fatty acids up to a pH of 4.3 and the denominator, partial alkalinity (PA), expresses the alkalinity of bicarbonate ions up to a pH of 5.75. The calculation of alkalinity values is carried out in a laboratory by titration. When the IA/PA ratio is greater than 0.3, the anaerobic system is disturbed; when the ratio is less than 0.3, there is stability in the system [24].

The stability of the anaerobic system can also be monitored by calculating the volatile fatty acids/total alkalinity (VFA/TA) ratio. VFA concentration and total alkalinity are calculated. Total alkalinity is the sum of the alkalinity of the bicarbonate ions (HCO_3_^−^), the carbonate ions (CO_3_^2−^) and the alkalinity of the hydroxyl ions (OH^−^). When the VFA/TA ratio is less than 0.3–0.4, the system is stable; when the value is greater than 0.5, there is instability in the anaerobic system [24].

### 3.5. Organic Load Rate (OLR)

In AnMBRs treating municipal wastewater, organic loads ranging from 0.3 to 12.5 kgCOD/m^3^d have been applied. Fluctuations in the incoming organic load rate between 0.2–12.5 kgCOD/m^3^d have been shown not to affect the quality of the treated effluent. In addition, it has been shown that increasing the incoming organic load entails a linear increase in the biogas produced [12]. However, high organic load is associated with changes in pH value. Specifically, it may result in the accumulation of volatile fatty acids, an increase in acidogenic bacteria, a decrease in the pH value of the wastewater and the restriction of the growth of methanogenic bacteria, resulting in poor treated effluent quality [23].

### 3.6. Solids Retention Time (SRT) and Hydraulic Retention Time (HRT)

The SRT is the average retention time of the sludge produced in the AnMBR bioreactor required for the anaerobic microorganisms to hydrolyze the suspended and colloidal components of the incoming feed stream. A high residence time is required to ensure the removal of soluble COD, to produce a large amount of methane and to produce lesser amounts of sludge that conventional biological treatments [29]. Therefore, sludge management of AnMBR requires lower levels of energy and there are fewer problems related to its final disposal than are are encountered in the conventional activated sludge process.

The HRT is the time specified for the residence of the wastewater feed stream within the AnMBR. A short hydraulic residence time means a smaller volume bioreactor and therefore lower capital costs. For municipal wastewater, a short hydraulic retention time is desirable to reduce the size of the AnMBR reactor and the overall footprint of the process. In contrast, a high SRT is required to achieve the required level of treatment, especially in areas where ambient temperatures are low [30]. In general, AnMBR at ambient temperature is only possible when the SRT time is twice that of the SRT time applied to mesophilic temperatures [12].

Huang et al. evaluated the performance of the AnMBR system in terms of COD removal rate and methane production at different SRTs (30 days and 60 days, without sludge removal) and HRTs (12, 10, 8 h) and observed that in all three cases the COD removal rate was 97%. In the third case, where no sludge was removed, maximum biogas production of 0.056 m^3^CH_4_/kgMLSSd was observed [31].

Yoo et al. report that 0.049 gVSS/gBOD_5_ of sludge is produced and removed during the treatment of municipal wastewater in an AnMBR system, while in aerobic secondary treatment the amount of sludge produced and removed is 0.42 gVSS/gBOD_5_ [32].

Xiao et al. report that a long SRT (213 days) improves the removal of micro-pollutants, such as pharmaceuticals, since the main mechanism of removal of these substances is their biodegradation by microorganisms and subsequently adsorption into the sludge [33]. Dutta et al. report that out of the 29 pharmaceutical substances found in municipal wastewater, 28 (with the exception of diclofenac) showed a removal rate greater than 86% after treatment in an AnMBR system [34].

However, a high SRT time of more than 140 days can lead to severe blockage of the membranes and reduce the rate at which the permeate flows through the membrane. The relationship between SRT time and membrane blockage is complex and cannot be quantified [12]. To reduce membrane blockage, an innovative technology being studied is the application of rotating membranes [35]. In this technique, submerged membranes are fitted to axes and circular motions are induced by an electric motor which increase the tangential speed. The result is better filtration and less blockage.

### 3.7. Toxicity of Free Ammonia, Sulfate Ions and Metals

The presence of nitrogen is essential for the metabolism of anaerobic microorganisms. The amount of nitrogen remains constant within the AnMBR system and simply changes from organic to inorganic forms. When nitrogen is found in the form of ammonium ions, it provides stability to the system, but in the form of free ammonia it is likely to interfere with the biological activity. The high concentration of free ammonia in the anaerobic bioreactor is enhanced by the increase in pH and temperature due to the conversion of ammonium ions to ammonia. Ammonia concentrations higher than 150 mg/L can be toxic to methanogenic microorganisms.

Sulfur is also essential for the growth of microbial cells. It is used by anaerobic sulfur-reducing microbes and converted to hydrogen sulfide in the liquid phase, which is toxic. When the sulfide concentration is greater than 200 mg/L, toxicity problems occur.

The higher the COD that is fed to the AnMBR, the higher the production of CH_4_, and the higher the conversion of sulfate to hydrogen sulfide (H_2_S). When H_2_S leaves the liquid phase and concentrates inside the bioreactor in the gas phase, its toxicity is reduced. If the ratio of COD/SO_4_^2−^ is greater than 10, then there are no toxicity problems in the anaerobic system. There are ways to control the sulfide concentration and maintain it at the desired level, such as precipitation by adding iron salts, increasing the COD/SO_4_^2−^ ratio to enhance H_2_S release in the gas phase and increasing pH.

Some trace elements and minerals, such as cobalt, iron and nickel, are also essential for the growth of microorganisms. Metals such as chromium, nickel, copper and arsenic are characterized as particularly toxic to anaerobic treatment. The presence of metals in high concentrations is in specific industrial streams rather than in municipal wastewater. One way to remove them from the anaerobic system is to add sulfides to form insoluble metal sulfides and cause precipitation [24].

## 4. Review of the Performance of AnMBRs in Municipal Wastewater Treatment

There are several published works in the international literature investigating the performance of AnMBR technology in municipal wastewater management. Table 1 presents important surveys of the last decade, namely, the period 2009–2021. Research is conducted on a pilot and laboratory scale and aims to investigate the performance and optimization of the system in terms of:

- Operating parameters, namely, temperature, pH, HRT and SRT;
- Biogas production;
- Fouling of the membranes;
- Removal of pollutants, such as COD, total organic carbon (TOC) and total suspended solids (TSS);
- The removal of nutrients, such as total nitrogen (TN) and total phosphorus (TP);
- The removal of organic micro-pollutants, such as endocrine disrupters, pharmaceuticals and substances contained in personal care products.

As an incoming load for treatment, untreated or pretreated municipal wastewater, domestic wastewater or synthetic wastewater, which simulates domestic wastewater, have been used. In all cases, it is the treatment of “weak” wastewater in terms of soluble organic content. For synthetic urban wastewater, the COD removal rate is most of the time greater than 95%, since all of the COD is soluble. Smith et al. [36] investigated the performance of the AnMBR system in terms of COD removal rate, using synthetic wastewater of a specific organic load in the first case and retaining the same operating parameters in the anaerobic system using untreated municipal wastewater. In the first case, the removal rate of the organic material was 92 ± 5%, while in the second case, it was 69 ± 10%. In investigations, the CSTR bioreactor is usually selected because of its ease of use and its in-house construction. The use of the UASB bioreactor is also common. In most cases, the performance and optimization of the AnMBR system with immersed membranes is investigated.

**Table 1:** Important scientific publications in municipal wastewater treatment with the AnMBR system.

#	Reactor Type/Membrane Configuration	Type of Membrane	Type of Wastewater	Flux (L/m^2^ × h)	Inlet COD (mg/L)	Operating Conditions T, pH, HRT	Outlet COD (mg/L)	COD Removal (%)	CH_4_ Produced	Ref.
	Year 2009									
1	Submerged CSTR	Flat sheet Polyethylene Pore size: 0.4 μm Area: 0.1 m^2^	Synthetic municipal wastewater	5–10	465	35 °C HRT = 8–20 h SRT = 250 d		99		[37]
2	UASB External	Tubular Polyacrylonitrile Area: 0.2 m^2^	Municipal wastewater	10.5	185.6	Ambient temp. HRT = 5.5–10 h OLR = 0.3–0.9 kgCOD/m^3^d		77–81	0.062 m^3^CH_4_/kgCOD	[38]
3	Submerged UASB	Tubular Polyethylene terephthalate Pore size: 0.64 μm Area: 0.98 m^2^	Municipal wastewater	5	259.5	15–20 °C HRT = 2.6 h OLR = 2.36 kgCOD/m^3^d	77.5 ± 29.5			[39]
4	UASB External	Flat sheet Polyvinylidene fluoride Pore size: 0.22 μm Area: 0.05 m^2^	Synthetic municipal wastewater	25	150 (TOC)	35 °C HRT = 12 h, 6 h, 4.5 h OLR = 0.3 kgCOD/m^3^d				[40]
5	External UASB	Hollow fiber Pore size: 0.2 μm Area: 4 m^2^	Pre-treated wastewater	3.75–11.25	540	25 °C HRT = 5.5–10 h OLR = 1.08–4.32 kgCOD/m^3^d		88		[4]
6	External CSTR	Tubular Ceramic (Al_2_O_3_) Pore size: 0.2 μm Area: 0.013 m^2^	Synthetic wastewater	20–40	10	55 °C SRT = 120 d OLR = 10–55 kgCOD/m^3^d				[41]
7	External AnMBR		Synthetic wastewater		500	25 °C SRT = 90–360 d OLR = 1 kgCOD/m^3^d HRT = 12 h OLR = 1.5 kgCOD/m^3^d HRT = 8 h OLR = 2 kgCOD/m^3^d HRT = 6 h	40	94	0.22 m^3^CH_4_/kgCOD 48% CH_4_ recovered 48–35% CH_4_ recovered 35% CH_4_ recovered	[15]
	**Year 2010**									
8	External CSTR	Pore size: 0.1 μm Area: 0.1 m^2^	Pre-treated diluted municipal wastewater	-	38–131	25 °C pH = 6.4 ± 0.2 HRT = 12–48 h SRT = 19–217 d OLR = 0.03–0.11 kgCOD/m^3^d	18–37	55–69		[42]
9	External CSTR	Tubular Polytetrafluoroethylene Pore size: 1 μm Area: 0.09 m^2^	Pre-treated sludge diluted with tap water	5	500	25 °C HRT = 12 h pH = 6.8–7.1 OLR = 1 kgCOD/m^3^d		95		[43]
	External CSTR	Tubular Polytetrafluoroethylene Pore size: 1 μm Area: 0.09 m^2^	Pre-treated sludge diluted with tap water	5	500	15 °C HRT = 12 h pH = 6.8–7.1 OLR = 1 kgCOD/m^3^d		85		[43]
10	External UASB	Tubular Pore size: 40 kDa Area: 81 cm^2^	Municipal wastewater	<7	646 ± 103	25 °C HRT = 3 h SRT = 100 d	104 ± 12	87		[44]
11	External UASB	Flat sheet Polyvinylidene fluoride Pore size: 100 μm Area: 0.052 m^2^	Synthetic wastewater	8–12	500	30 °C HRT = 24 h SRT = 50 d OLR = 5 kgCOD/m^3^d		96		[45]
12	External CSTR	Hollow fiber Pore size: 0.2 μm Area: 4 m^2^	Municipal wastewater	7.5	540	25 °C HRT = 6 h OLR = 2.16 kgCOD/m^3^d	65	88		[4]
13	External CSTR	Flat sheet Pore size: 0.4 μm Area: 0.12 m^2^	Synthetic	2–5		35 ± 1 °C SRT = 30–40 d OLR = 1.5–13 kgCOD/m^3^d	53 g/L TSS	60–80		[46]
14	Submerged CSTR	Hollow fiber Polypropylene Pore size: 0.45 μm Area: 0.003 m^2^	Synthetic			35 °C SRT = 14 d OLR = 2.5 kgCOD/m^3^d		99.6		[47]
	**Year 2011**									
15	External CSTR	Hollow fiber Area: 5.4 m^2^	Pre-treated municipal wastewater	17	224	22 °C HRT = 8.5 h SRT = 80–100 d OLR = 0.58 kgCOD/m^3^d	47	79		[48]
16	External CSTR	Flat sheet Polyethersulfone Pore size: 38 nm Area: 3.5 m^2^	Pre-treated municipal wastewater (with glucose addition)	7	630 ± 82	35 °C HRT = 19.2 h SRT = 680 d OLR = 0.5–0.9 kgCOD/m^3^d	80	90	0.27 m^3^CH_4_/kgCOD	[21]
17	Submerged CSTR	Flat sheet Polyethersulfone Pore size: 38 nm Area: 3.5 m^2^	Pre-treated municipal wastewater (with glucose addition)	7	630 ± 82	20 °C HRT = 19.2 h SRT = 680 d OLR = 0.5–0.9 kgCOD/m^3^d		82	0.23 m^3^CH_4_/kgCOD	[21]
18	Submerged CSTR	Flat sheet Polyolefin Pore size: 0.4 μm Area: 0.1 m^2^	Synthetic wastewater	10.5	425	HRT = 12 h		83	-	[49]
19	Submerged CSTR	Hollow fiber Pore size: 0.05 μm Area: 30 m^2^	Municipal wastewater	10	445	HRT = 6–20 h SRT = 70 d		87	0.069 m^3^CH_4_/kgCOD	[50]
20	Submerged AnMBR	Flat sheet Polyethersulfone Pore size: 0.2 μm	Synthetic municipal wastewater		440	15 °C HRT = 16 h SRT = 300 d	36	92		[51]
21	Submerged CSTR	Flat sheet Polyvinylidene fluoride Pore size: 140 kDa Area: 0.6 m^2^	Municipal wastewater	12	302.1 ± 87.9	30 °C pH = 7.6 ± 0.3 HRT = 10 h OLR = 1 kgCOD/m^3^d	51 ± 10	88 ± 2	0.24 m^3^CH_4_/kgCOD	[52]
22	Submerged CSTR	Flat sheet Polyethersulfone Pore size: 0.45 μm Area: 0.118 m^2^	Synthetic municipal wastewater		550	25–30 °C SRT = 30–60 d HRT = 8 h	17	97		[31]
23	External CSTR (two-stage)	Tubular Ceramic Pore size: 0.1 μm	Synthetic wastewater	-	10,200–23,900	55 °C pH = 5.5–7.2 HRT = 48 h OLR = 5.1–12 kgCOD/m^3^d		78–81		[28]
24	External UASB	Tubular Polyvinylidene fluoride Pore size: 100 kDa	Synthetic municipal wastewater		350	HRT = 4 h	70	80		[53]
25	Two-stage fluidized bed submerged membrane	Hollow fiber Polyvinylidene fluoride Pore size: 0.1 μm	Synthetic municipal wastewater		513	35 °C HRT = 4.2–5.9 h	7	99		[16]
	**Year 2012**									
26	Submerged CSTR	Hollow fiber Pore size: 0.05 μm Area: 30 m^2^	Pre-treated municipal wastewater		410	33 °C HRT = 6–21 h SRT = 76 d OLR = 0.71 kgCOD/m^3^d			Recovery 57.4% CH_4_	[54]
27	Submerged CSTR	Hollow fiber Pore size: 0.05 μm Area: 30 m^2^	Pre-treated municipal wastewater		720	21 °C HRT = 6–21 h SRT = 74 d OLR = 0.64 kgCOD/m^3^d			Recovery 53.6% CH_4_	[54]
28	Gas sparging AnMBR submerged	Hollow fiber Pore size: 0.05 μm Area: 30 m^2^	Municipal wastewater	9–13.3		17–33 °C HRT = 6–26 h OLR = 0.3–1.1 kgCOD/m^3^d pH = 6.5–7.1		85		[55]
	**Year 2013**									
29	Submerged UASB	Hollow fiber Polyvinylidene fluoride Pore size: 0.1 μm Area: 1 m^2^	Synthetic wastewater	5	500 ± 10	27 °C HRT = 2 h 38 gCOD/gMLSSd OLR = 6 kgCOD/m^3^d		>90	0.637 ± 0.213 m^3^CH_4_/kgMLSSd	[56]
30	Submerged UASB	Hollow fiber Polyvinylidene fluoride Pore size: 0.1 μm Area: 1 m^2^	Synthetic wastewater	5	500 ± 10	30 °C HRT = 12 h OLR = 0.1 gCOD/gMLSSd OLR = 1 kgCOD/m^3^d		>90	0.406 ± 0.101 m^3^CH_4_/kgMLSSd	[56]
31	Gas-lift AnMBR submerged	Tubular Polyvinylidene fluoride Pore size: 0.03 μm Area: 0.013 m^2^	Synthetic municipal wastewater	10–15	COD = 1260 TN = 54 TP = 44			95–98	4.5 L/d	[57]
32	Bench scale AnMBR submerged	Flat sheet Polyethersulfone Pore size: 0.2 μm Area: 0.0387 m^2^	Synthetic municipal wastewater	7	227 (BOD_5_)	15 °C	COD = 43 BOD_5_ = 18	92	40–50% CH_4_ recovered	[36]
33	Bench scale AnMBR submerged	Flat sheet Polyethersulfone Pore size: 0.2 μm Area: 0.0387 m^2^	Municipal wastewater	7		15 °C	COD = 76 BOD_5_ = 24	69		[36]
	**Year 2014**									
34	Two-stage fluidized reactor fed with membrane submerged in an external tank	Hollow fiber Polyvinylidene fluoride Pore size: 0.03 μm Area: 0.004 m^2^	Municipal wastewater	1–7.2	198–285 TSS: 67 ± 13	9–25 °C HRT = 6.1 h	14–28	93		[58]
35	External CSTR	Flat sheet Pore size: 200 μm	Synthetic municipal wastewater (sucrose)		5000	35 °C HRT = 1–7 d OLR = 0.7–5 gCOD/m^3^d	300–1800	75		[59]
36	Two-stage fluidized fed with membrane submerged in an external tank	Tubular Polyvinylidene fluoride Pore size: 0.1 μm	Municipal wastewater		235–300	10–25 °C HRT: 2.3 h	21–37	>86		[60]
37	Two-stage AFBR–AFMBR with submerged membranes	Hollow fiber Polyvinylidene fluoride Pore size < 0.1 μm Area: 0.04 m^2^	Synthetic wastewater		250–1000	20–25 °C HRT = 1.28 h	10	>90		[34]
38	Submerged UASB	Hollow fiber Pore size: 0.04 μm Area: 0.0245 m^2^	Synthetic wastewater	10	240 ± 15	30 ± 1 °C pH = 7.5 HRT = 6 h SRT = 60 d		89 (TOC)		[61]
39	Gas sparging AnMBR submerged	Flat sheet Cellulose triacetate Area: 0.025 m^2^	Synthetic municipal wastewater	3.5–9.5		25 °C HRT = 15–40 h		97	0.21 m^3^CH_4_/kgCOD	[62]
40	Granular activated carbon AnMBR–UASB submerged	Hollow fiber Pore size: 0.4 μm Area: 0.19 m^2^	Synthetic municipal wastewater	11.3	247–449	15–35 °C HRT = 6 h OLR = 1.21–1.44 kgCOD/m^3^d pH = 7.18–7.99		51–74	0.14–0.19 m^3^CH_4_/kgCOD	[63]
41	AnMBR side-stream	Hollow fiber Polyvinylidene fluoride Pore size: 0.03 μm Area: 0.031 m^2^	Synthetic municipal wastewater		400	35 °C HRT = 6–12 h OLR = 0.8–10 kgCOD/m^3^d pH = 7		97–99	0.088–0.393 m^3^CH_4_/kgCOD	[64]
	**Year 2015**									
42	Submerged AnMBR in an external tank	Hollow fiber Pore size: 0.05 μm Area: 30 m^2^	Pre-treated municipal wastewater	19	380	15 °C pH = 7 alkalinity = 350 mgCaCO_3_/L HRT = 14 h SRT = 35 d OLR = 0.5–2 kgCOD/m^3^d 30 °C pH = 7 Alkalinity = 350 mgCaCO_3_/L HRT = 7 h SRT = 12 d OLR = 0.5–2 kgCOD/m^3^d	BOD = 25			[6]
43	Submerged AnMBR in an external tank	Hollow fiber Pore size: 0.05 μm Area: 30 m^2^	Pre-treated municipal wastewater	19	380	15 °C pH = 7 350 mgCaCO_3_/L HRT = 23 h SRT = 60 d OLR = 0.5–2 kgCOD/m^3^d 30 °C pH = 7 350 mgCaCO_3_/L HRT = 10 h SRT = 22 d OLR = 0.5–2 kgCOD/m^3^d	BOD = 25			[6]
44	CSTR submerged in an external tank	Hollow fiber Polyvinylidene fluoride Pore size: 0.04 μm	Municipal wastewater		252 ± 59	23 ± 1 °C pH = 6.8 HRT = 8.5 h	17–29	90		[65]
45	External CSTR	Ceramic Pore size: 80, 200, 300 nm	Municipal wastewater		330.4 ± 89.8	25–30 °C HRT = 7.5 h SRT = 60 d		86–88	0.1 ± 0.02 m^3^CH_4_/kgCOD	[66]
46	External UASB	Tubular Pore size: 30 μm Area: 0.11 m^2^	Synthetic wastewater	12.3	530 ± 30	25 °C HRT = 6 h SRT = 126 d OLR = 2 kgCOD/m^3^d	42	92		[67]
47	External UASB	Tubular Pore size: 30 μm Area: 0.11 m^2^	Synthetic wastewater	12.3	530 ± 30	15 °C HRT = 6 h SRT = 126 d OLR = 2 kgCOD/m^3^d	52	90		[67]
48	External UASB	Tubular Pore size: 30 μm Area: 0.0038 m^2^	Synthetic wastewater	12.3	530 ± 30	15–25 °C HRT = 6 h SRT = 126 d OLR = 2 kgCOD/m^3^d	149 ± 5.9 42 ± 4.4	92	-	[68]
49	External UASB	Tubular Pore size: 0.045 μm Area: 0.93 m^2^	Pre-treated municipal wastewater (passing through 1 mm sieve)		892 ± 271	18 ± 2 °C HRT = 7 h	100–120	87 ± 1	63.8 ± 15.7 L/d	[69]
50	Gas sparging AnMBR–UASB submerged	Hollow fiber Polyvinylidene fluoride Pore size: 0.045 μm Area: 0.93 m^2^	Municipal wastewater	8–15	978	18 °C HRT = 9.8–20.3 h OLR = 0.6–3.18 kgCOD/m^3^d pH = 7.2		75–90	0.26–0.14 m^3^CH_4_/kgCOD	[70]
51	Gas sparging AnMBR submerged	Flat sheet Cellulose triacetate Surface Area: 0.025 m^2^	Synthetic municipal wastewater	3–10		35 °C HRT = 15–40 h pH = 7		>95	0.25–0.3 m^3^CH_4_/kgCOD	[71]
	**Year 2016**									
52	Semi-fluidized bed membrane submerged in an external tank	Tubular Ceramic Pore size: 0.05 μm Area: 0.05 m^2^	Pre-treated synthetic + real municipal watewater	5.3	480 ± 50	10–25 °C HRT: 4.2–9.8 h	<26 ± 15	>94		[72]
53	Downflow floating filter (DFF) membrane submerged in an external tank	Tubular Polyvinylidene fluoride Pore size: 100 kDa Area: 0.059 m^2^	Pre-treated synthetic + real municipal watewater	5.3	480	10–25 °C HRT = 6–14 h	<25	>95		[73]
54	Submerged AnMBR	Flat sheet Polyvinylidene fluoride Pore size: 0.2 μm Area: 0.735 m^2^	Municipal wastewater		400	25 °C and 35 °C HRT = 5.8–4.8 h HRT = 8–7.1 h SRT = 50 d OLR = 0.43–0.90 kgCOD/m^3^d	-	90		[2]
55	External CSTR	Hollow fiber Polyvinylidene fluoride Pore size: 30 nm Area: 320 cm^2^	Synthetic wastewater	6	400 ± 10	35 ± 1 °C pH = 7 ± 0.1 HRT = 12 h	20	97	0.25 m^3^CH_4_/kgCOD	[74]
56	External CSTR	Flat sheet Polyvinylidene fluoride Pore size: 200 kDa Area: 0.735 m^2^	Synthetic wastewater	6	800	35 ± 1 °C pH = 7 ± 0.1 HRT = 12 h (addition of 100 mg/L activated carbon)		99		[74]
	**Year 2017**									
57	External UASB	Hollow fiber Polyvinylidene fluoride Pore size: 0.22 μm	Synthetic municipal wastewater		330–370	20 ± 0.5 °C pH = 7.0 HRT = 12 h	26.6–30	91.9		[75]
58	Submerged UASB	Hollow fiber Polyvinylidene fluoride Pore size: 0.22 μm	Synthetic municipal wastewater		330–370	20 ± 0.5 °C pH = 7 HRT = 12 h	28.7–32.2	91.3		[75]
59	Two-stage anaerobic semi-fluidized bed submerged membrane	Hollow fiber Pore size: 0.1 μm Area: 0.022 m^2^	Primary effluent (clarifier)	30	70	23 ± 1 °C HRT = 4 h pH = 7.2	24	>97	2.11 L/d	[76]
60	One-stage anaerobic semi-fluidized bed submerged membrane	Hollow fiber Pore size: 0.1 μm Area: 0.022 m^2^	Primary effluent (clarifier)	30	48	23 ± 1 °C HRT = 3 h pH = 7.3	18	>97	2.11 L/d	[76]
61	Submerged UASB	Tubular Polyvinylidene fluoride Area: 0.2375 m^2^	Municipal wastewater	2.5	525 ± 174 657 ± 235	18–21 °C HRT = 8 h	222 ± 61 130 ± 55	68.6	-	[77]
62	Conventional granular AnMBR submerged membrane	Hollow fiber Polyvinylidene fluoride Pore size: 0.22 μm Area: 0.06 m^2^	Synthetic wastewater	5.3	330–370	20 °C HRT = 12 h SRT = 25–30 d MLSS = 20.50 ± 1.53 g/L	-	90.8 ± 1.4	0.1333 ± 0.0053 m^3^CH_4_/kgCOD	[75]
63	Sponge granular AnMBR submerged membrane	Hollow fiber Polyvinylidene fluoride Pore size: 0.22 μm Area: 0.06 m^2^	Synthetic wastewater	5.3	330–370	20 °C HRT = 12 h SRT = 25–30 d MLSS = 20.50 ± 1.53 g/L		93.7 ± 1.7	0.1563 ± 0.0058 m^3^CH_4_/kgCOD	[78]
64	Submerged AnMBR	Flat sheet Ceramic Pore size: 80 nm Area: 0.08 m^2^	Municipal wastewater	8		25 °C HRT = 5.8 h SRT = 60 d OLR = 10 kgCOD/m^3^d	417 ± 61	87	-	[79]
65	Submerged AnMBR	Flat sheet Pore size: 0.2 μm Area: 0.116 m^2^	Synthetic municipal wastewater (contains alcohol ethoxylates used as personal care products)	-	492 ± 112	25 ± 1 °C HRT = 42–12 h OLR = 3–6 kgCOD/m^3^d	17.1	95.5–98.8	2.30–4.25 L/d	[80]
66	Submerged AnMBR	Flat sheet Ceramic (alumina)	Synthetic municipal wastewater	3.3 ± 0.21	600–800	HRT = 18 ± 1.3 h	-	96.1 ± 5.1		[81]
67	Submerged AnMBR	Flat sheet Ceramic (pyrophyllite)	Synthetic municipal wastewater	2.7 ± 0.12	600–800	HRT = 18 ± 1.6 h	-	42.6 ± 19.2	0.16 m^3^CH_4_/kgCOD	[81]
68	CSTR membrane submerged in external tank	Flat sheet Pore size: 0.2 μm Area: 5.4 m^2^	Pre-treated municipal wastewater	6	223 ± 111	35 °C HRT = 2.2 h SRT = 60 d OLR = 3 kgCOD/m^3^d	50 ± 22	87	0.12 m^3^CH_4_/kgCOD	[82]
69	External CSTR	Hollow fiber Polyvinylidene fluoride Pore size: 0.04 μm Area: 0.9 m^2^	Municipal wastewater	22.5	1.462 ± 693	18.9 °C HRT = 33 h SRT = 270 d OLR = 1.1 kgCOD/m^3^d	129 ± 55	91	0.012 m^3^CH_4_/kgMLVSSd	[35]
70	Gas-lift AnMBR external	Tubular Polyvinylidene fluoride Pore size: 0.03 μm Area: 0.066 m^2^	Synthetic municipal wastewater	4.22–4.37		35 °C + shocks 15 °C	55 ± 18	94 ± 2	0.19 m^3^CH_4_/kgCOD	[22]
71	External AnMBR	Polyvinylidene fluoride Pore size: 0.3 μm	Synthetic enriched with three types of bacteria resistant to antibiotics	7	COD = 750 Bacteria = 2.76–3.84 log units	35 °C pH = 7 HRT = 11 SRT = 700 d OLR = 0.43 kgCOD/m^3^d		>93		[83]
72	AnMBR—submerged coupled with activated carbon	Flat sheet Pore size: 0.2 μm Area: 0.11 m^2^	Synthetic with five pharmaceutical substances	5	COD = 500	35 °C HRT = 6 h SRT = 213 d		93.8	1.8 ± 0.3L/d	[33]
73	Gas-sparging AnMBR submerged	Flat sheet Polyvinylidene fluoride Pore size: 0.2 μm Area: 0.025 m^2^	Synthetic municipal wastewater	2–6	372.6	25 °C HRT = 35–60 h		90–96	0.25–0.28 m^3^CH_4_/kgCOD	[84]
74	Gas-sparging AnMBR submerged	Hollow fiber Pore size: 0.2 μm Area: 5.4 m^2^	Municipal wastewater	6		35 °C HRT = 2.2 h OLR = 3 kgCOD/m^3^d		87	0.12 m^3^CH_4_/kgCOD	[82]
	**Year 2018**									
75	AnMBR submerged sludge recirculation	Flat sheet Pore size: 75 μm Area: 0.02 m^2^	Pretreated (i) municipal (ii) municipal and synthetic (iii) municipal + high strength wastewater	22.5	(i) 292 (ii) 516 (iii)1028	20 °C HRT = 8 h (i) OLR = 0.88 kgCOD/m^3^d (ii) OLR = 1.55 kgCOD/m^3^d (iii) OLR = 3.01 kgCOD/m^3^d	(i) 77.5 ± 19.2 (ii)108.4 ± 45.9 (iii) 82.5 ± 30.9	92	(Ι) 0.25 ± 0.08 L/d (ΙΙ) 0.37 ± 0.13 L/d (ΙΙΙ) 1.65 ± 0.45 L/d	[85]
76	AnMBR submerged	Hollow fiber Polyvinylidene fluoride Pore size:0.22 μm Area: 0.04 m^2^	Synthetic municipal wastewater		300	HRT = 18–12 h SRT = 35 d	-	95 (TOC)	-	[86]
77	Sponge-AnMBR submerged	Hollow fiber Polyvinylidene fluoride Pore size: 0.22 μm Area: 0.04 m^2^	Synthetic municipal wastewater	-	300	HRT = 18–12 h SRT = 35 d	-	95 (TOC)	-	[86]
78	Anaerobic fluidized bed external	Tubular Ceramic Pore size: 0.05 μm	Synthetic		235–160	20 °C HRT = 6 h SRT = 49 d	10	>90		[87]
79	AnMBR external	Tubular Polyvinylidene fluoride Pore size: 20 kDa Area: 0.011 m^2^	Synthetic municipal wastewater	165	COD = 445 NH_4_-N = 42	HRT = 37.5 h OLR = 0.25 kgCOD/m^3^d	39	91		[88]
80	AnMBR external	Tubular Polyvinylidene fluoride Pore size: 20,000 Area: 0.011 m^2^	Synthetic municipal wastewater	165	562 NH_4_-N = 51	HRT = 13 h OLR = 0.7 kgCOD/m^3^d	31	94		[88]
81	AnMBR submerged	Hollow fiber Pore size: 46.5 nm	Municipal		350 500 650 750	25 °C			110 LCH_4_/m^3^ 157 LCH_4_m^3^ 204 LCH_4_/m^3^ 236 LCH_4_/m^3^	[89]
82	AnMBR submerged	Hollow fiber Pore size: 46.5 nm	Municipal					7.3		[90]
83	Gas-sparging AnMBR–UASB submerged	Hollow fiber Polyvinylidene fluoride Pore Size: 0.04 μm Area: 0.93 m^2^	Municipal	9–15	221	16.3 °C HRT = 8 h pH = 7.8		83		[91]
84	UASB submerged	Hollow fiber Pore size: 0.045 μm Area: 0.93 m^2^	Municipal	6–7 (10 °C) 10–12 (28 °C)	372± 149	28–10 °C HRT = 8–10 h pH = 8.2 ± 0.3 OLR = 1 kgCOD/m^3^d	150	89	0.09–0.14 Nm^3^CH_4_/kgCOD	[92]
	**Year 2019**									
85	Two-stage anaerobic fluidized bed submerged	Polyvinylidene fluoride Pore size: 0.1 μm Area: 0.255 m^2^	Synthetic municipal + granular activated carbon	1.8	150	25 °C HRT = 8.72 h	26.5 ± 20.7	96.2 ± 1.6	-	[93]
86	CSTR external	Hollow fiber Pore size: 0.40 μm Area: 0.073 m^2^	Synthetic	(i) 10.3 (ii) 8.8 (iii) 6.0	500	HRT = 26.2 h pH = 6.8–7.2 alkalinity = 2187 mgCaCO_3_/L OLR = 0.46 kgCOD/m^3^d	17.7	96.7	0.44 m^3^ biogas/kgCOD	[94]
87	AnMBR external	Flat sheet Polyvinylidene fluoride Pore size: 0.3 μm	Synthetic	6	800	35 °C pH = 7 HRT = 44 h SRT = 1400 d OLR = 0.43 kgCOD/m^3^d		96.6	0.2313 m^3^CH_4_/kgCOD	[95]
88	AnMBR external	Flat sheet Polyvinylidene fluoride Pore size: 0.3 μm	Synthetic	6	800	35 °C pH = 7 HRT = 22 h SRT = 700 d OLR = 0.86 kgCOD/m^3^d		96.2	0.2199 m^3^CH_4_/kgCOD	[95]
89	AnMBR submerged	Hollow fiber Pore size: 0.07–0.1 μm Area: 0.08 m^2^	Synthetic		550	22 °C HRT = 8 h (i) pH = 5 (ii) pH = 6 (iii) pH = 7 (iv) pH = 8 (v) pH = 10 (vi) pH = 12		(i) 60.5 (ii) 63.4 (iii) 79.8 (iv) 76 (v) 75 (vi) 68.5		[96]
90	AnMBR submerged	Hollow fiber Pore size: 0.07–0.1 μm Area: 0.08 m^2^	Synthetic wastewater		550	22 °C pH = 7 (i) HRT = 48 h (ii) HRT = 24 h (iii) HRT = 18 h (iv) HRT = 12 h (v) HRT = 8 h (vi) HRT = 6 h		70		[97]
91	AnMBR submerged	Hollow fiber Pore size: 0.07–0.1 μm Area: 0.08 m^2^	Synthetic wastewater		(i) 350 (ii) 550 (iii) 715	22 °C pH = 7 HRT = 8 h		(i) 70.9 (ii) 70.9 (iii) 65.1		[97]
92	AnMBR submerged	Flat sheet Polyethersulfone Pore size: 0.2–0.4 μm Area: 0.1 m^2^	Synthetic wastewater + antibiotics Ciprofloxacin (CIP)		500	HRT = 12 h–6 h SRT = 300 d	14–24	>95		[98]
93	AnMBR submerged	Flat sheet Polyethersulfone Pore size: 0.2–0.4 μm Area: 0.1 m^2^	Synthetic wastewater + Ciprofloxacin		500	HRT = 12 h–6 h SRT = 300 d		78		[98]
94	AnMBR submerged	Flat sheet Polyethylene Pore size: 0.2–0.4 μm Area: 0.1 m^2^	Synthetic wastewater		500	HRT = 12 h–6 h SRT = 300 d		89 ± 2		[98]
95	Gas-sparged AnMBR submerged	Hollow fiber Polyvinylidene fluoride Pore size: 0.04 μm	Municipal wastewater (after screening)	7.7	620 ± 240	13–32 °C HRT = 11 ± 3 h OLR = 1.3 kgCOD/m^3^d	58 ± 27	90		[99]
96	GAC-fluidized AnMBR submerged	Hollow fiber Polyvinylidene fluoride Pore size: 0.03 μm	Municipal wastewater (after screening)	8	210 ± 50	13–32 °C HRT = 4 h OLR = 1.4 kgCOD/m^3^ d	29 ± 9	86		[99]
97	AnMBR submerged	Flat sheet Polyvinylidene fluoride Pore size: 0.1 μm Area 0.08 m^2^	Synthetic wastewater			35 °C HRT = 6 h SRT = 120 d	<20	96.4	0.36–0.42 m^3^biogas/kgCOD	[100]
98	AnMBR submerged	Flat sheet Polyvinylidene fluoride Pore size: 0.1 μm Area: 0.08 m^2^	Synthetic wastewater + ZnO NPs (0.4 mg/L/d)			35 °C HRT = 6 h SRT = 120 d	33	81.5	0 m^3^ biogas/kgCOD (Inhibition by Zn^+2^)	[100]
99	AnMBR submerged	Hollow fiber Polyvinylidene fluoride Pore size: 0.2 μm Area: 0.2 m^2^	Synthetic wastewater	1.68	(i) 570 (ii) 630 (iii) 578	35 °C (i) HRT = 24 h (ii) HRT = 12 h (iii) HRT = 6 h	(i) 50 (ii) 40 (iii) 44	(i) 91 (ii) 93 (iii) 92		[101]
100	AnMBR submerged	Hollow fiber Polyvinylidene fluoride Pore size: 0.2 μm Area: 0.2 m^2^	Synthetic wastewater	1.68	(i) 570.41 (ii) 630.49 (iii) 578.15	25 °C (i) HRT = 24 h (ii) HRT = 12 h (iii) HRT = 6 h	(i) 137 (ii) 109 (iii) 170	(i) 91 (ii) 93 (iii) 92		[101]
101	AnMBR external	Hollow fiber Polyvinylidene fluoride Pore size: 0.03 μm Area: 0.031 m^2^	Synthetic wastewater + Sulfamethoxazole	3	810	35 °C HRΤ = 24 h pH = 7	25.2	96.9	0.0813 m^3^CH_4_/kgCOD	[102]
102	AnMBR submerged	Hollow fiber Pore size: 0.1 μm Area: 0.007 m^2^	Synthetic wastewater	8	500	35 ± 1 °C HRΤ = 18.5 h	25	96.1	0.255–0.318 m^3^CH_4_/kgCOD	[103]
	**Year 2020**									
103	Biochar-amended AnMBR	Hollow fiber Polyvinylidene fluoride Pore size: 0.02 μm Area: 0.1 m^2^	Pharmaceutical wastewater			32 °C HRT = 24 h OLR = 7 kgCOD/(m^3^d)		93.8 ± 1.7		[104]
104	AnMBR submerged	Hollow fiber Polyvinylidene fluoride Pore size: 0.04 μm Area: 0.146 m^2^	Municipal wastewater	i. 5.71 ii. 11.42 iii. 9.50 iv. 11.42	408	25.2 °C pH = 7.3 (i) HRT = 24 h (ii) HRT = 12 h (iii) HRT = 14.4 h (iv) HRT = 12 h	(i) 53.6 (ii) 42.1 (iii) 39.0 (iv) 44.0	(i) 88.9 (ii) 89.8 (iii) 89.0 (iv) 88.1	i. 0.15 m^3^CH_4_/kgCOD ii. 0.15 m^3^CH_4_/kgCOD iii. 0.18 m^3^CH_4_/kgCOD iv. 0.19 m^3^CH_4_/kgCOD	[105]
105	AnMBR submerged	Hollow fiber Polyvinylidene fluoride Pore size: 0.05 μm Area: 0.270 m^2^	Municipal wastewater	i. 3.08 ii. 5.13 iii. 6.17	408	25.2 °C pH = 7.3 (i) HRT = 24 h (ii) HRT = 14.4 h (iii) HRT = 12 h	(i) 47.1 (ii) 42.8 (iii) 41.6	(i) 88.9 (ii) 88.9 (iii) 89.5	i. 0.16 m^3^CH_4_/kgCOD ii. 0.2 m^3^CH_4_/kgCOD iii. 0.18 m^3^CH_4_/kgCOD	[105]
106	AnMBR external	Ceramic Pore size: 0.4 μm Area: 0.08 m^2^	Synthetic wastewater + 15 trace organic contaminants	0.94	2152.9 (TOC)	35 °C pH = 6.96 HRT = 48 h		98 (TOC)	0.277 m^3^CH_4_/kgCOD	[106]
107	Anaerobic fluidized bed membrane bioreactor submerged	Flat-tubular Ceramic (Al_2_O_3_) Pore size: 0.1 μm Area: 0.09 m^2^	Synthetic wastewater	10.4	300.1	35 °C pH = 7.5 HRT = 8 h OLR = 0.9 kgCOD/m^3^d	30.1	90.0	0.216 m^3^CH_4_/kgCOD	[107]
108	AnMBR submerged		Low-strength domestic sewage		269–712	32 °C HRT = 6–22 h OLR = 0.29–2.85 kgCOD/m^3^d pH = 6.98–7.19		64.41–83.49		[108]
109	AnMBR submerged	Polyvinylidene fluoride Pore size: 0.1 μm Area: 0.1029 m^2^	2-chlorophenol synthetic wastewater	2.02–4.04	560–2200	36 °C HRT = 48–96 h OLR = 0.28–1.12 kgCOD/m^3^d		93.2		[109]
110	Sponge-based moving bed-anaerobic osmosis membrane bioreactor/membrane distillation (AnOMBR/MD) system	Tubular forward osmosis membrane Cellulose triacetate Area: 120 cm^2^ Polyvinylidene fluoride Pore Size: 0.45 μm Membrane distillation Pore Size: 0.45 μm Area: 200 cm^2^	Municipal wastewater	4.01	880–1120	45 °C HRT = 40–50 h pH = 7.3	<5	>99	0.11–0.18 m^3^CH_4_/kgCOD	[110]
111	AnMBR submerged	Flat Polyethersulfone Area: 0.034 m^2^	Model slurry of garbage waste from the food industry		23,233	35 °C HRT = 4 d OLR = 5.8 kgCOD/m^3^d pH = 7		>98		[111]
112	Two-stage AnMBR	Hollow fiber Polyvinylidene fluoride Pore size: 0.04 μm Area: 0.065 m^2^	Sugarcane vinasse pre-treated by ultrafiltration		18,777	22 °C HRT = 11.5 h (acidogenic reactor) HRT = 61.8 h (methanogenic reactor) pH = 5.2	2204	88.3		[112]
113	Anaerobic hybrid membrane bioreactor	Ceramic Pore size: 0.1 μm Area: 0.035 m^2^	Synthetic leachate	70–52	27,850	35 °C HRT = 48 h OLR = 13.9 kgCOD/m^3^d pH = 6.9	3261	88		[113]
	**Year 2021**									
114	Anaerobic osmotic membrane bioreactor	Forward osmosis Area: 70 cm^2^	Synthetic	5.78	4000	35 °C pH = 7.48		93.4	0.21 m^3^CH_4_/kgCOD	[114]
115	Granular activated carbon-synergized anaerobic membrane bioreactor	Hollow fiber Polyvinylidene fluoride Pore size: 0.1 μm Area: 20 m^2^	Municipal	16	277–348	5–35 °C HRT = 6–24 h pH = 6.8–7.3	<50	>86	0.24 m^3^CH_4_/kgCOD	[115]
116	AnMBR submerged	Hollow fiber Polyvinylidene fluoride Pore size: 0.4 μm Area: 72 m^2^	Municipal	2.75–17.83	362.2–481.9	25 °C HRT = 6–24 h OLR = 1.84 kgCOD/m^3^d pH = 6.91–7.2	29.2–42.9	89.5–93.2	0.25–0.27 m^3^biogas/kgCOD CH4 content 75–81%	[116]
117	AnMBR submerged	Hollow fiber Polyvinylidene fluoride Pore Size: 0.4 μm Area: (i–iii) 0.345 m^2^ (iv) 0.146 m^2^	Municipal	(i) 14.16 (ii) 9.61 (iii) 7.23 (iv) 11.02	i. 350 ii–iv. 365	(i–iv) 25 °C (i–iv) pH = 7.16 (i) HRT = 4 h OLR = 2.05 kgCOD/m^3^d (ii) HRT = 6 h OLR = 1.52 kgCOD/m^3^d (iii) HRT = 8 h OLR = 1.18 kgCOD/m^3^d (iv) HRT = 12 h OLR = 0.72 kgCOD/m^3^d		89	(i) 0.16 m^3^CH_4_/kgCOD (ii) 0.23 m^3^CH_4_/kgCOD (iii) 0.24 m^3^CH_4_/kgCOD (iv) 0.21 m^3^CH_4_/kgCOD	[117]
118	AnMBR submerged	Flat sheet Polyvinylidene fluoride Pore size: < 0.1 μm Area: 0.1 m^2^	Municipal	(i) 1.6 (ii) 1.6 (iii) 3.3 (iv) 3.3 (v) 6.6	(i) 477 (ii) 470 (iii) 456 (iv) 428 (v) 455	(i–vi) pH = 6.9–7.19 (i) 18 °C HRT = 48 h OLR = 0.225 kgCOD/m^3^d (ii) 23 °C HRT = 48 h OLR = 0.225 kgCOD/m^3^d (iii) 19 °C HRT = 24 h OLR = 0.45 kgCOD/m^3^d (iv) 24 °C HRT = 24 h OLR = 0.45 kgCOD/m^3^d (v) 19 °C HRT = 12 h OLR = 0.9 kgCOD/m^3^d	(i) 105 (ii) 51 (iii) 95 (iv) 67 (v) 123	(i) 76 (ii) 89 (iii) 77 (iv) 85 (v) 69	(i) 0.75 LCH_4_/d (ii) 0.56 LCH_4_/d (iii) 1.57 LCH_4_/d (iv) 1.12 LCH_4_/d (v) 3.14 LCH_4_/d	[118]

## 5. Evaluation of Results

**Figure 5:**
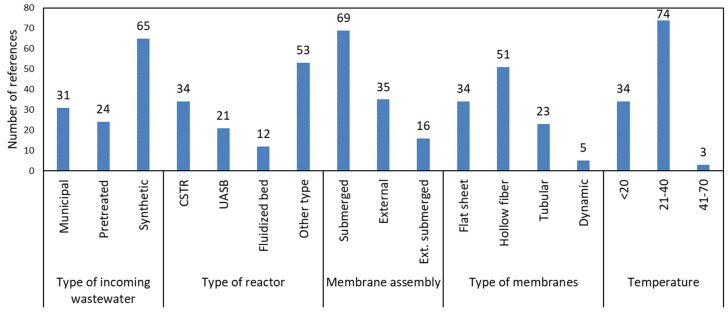
Type of incoming feed stream, type of the bioreactor, type of membrane configuration applied, membrane types used, the prevailing temperature conditions and COD removal distribution in anaerobic treatment.

All bibliographic references refer to pilot and laboratory studies. There is no documented full-scale AnMBR treating municipal/domestic wastewater. The results of the classification are shown in Figure 5.

### 5.1. Type of Incoming Wastewater

In 65 of the 118 investigations included in Table 1, synthetic wastewater was used to simulate the quality characteristics of municipal and domestic wastewater (Figure 5). The use of synthetic wastewater was preferred in most research as it is easier to produce and does not contain potentially inhibiting substances [15,53]. This ensures optimum system performance and therefore understanding of the operating parameters of anaerobic treatment and how the system performs most efficiently. It is also possible to study membrane fouling that appears to remain the major obstacle to the deployment and wide application of AnMBR technology [88]. Moreover, the use of synthetic wastewater over real wastewater allows for the investigation of the impact of emerging contaminants on anaerobic systems [102]. To address conditions that may adversely affect the proper conduct of anaerobic treatment, many studies indicate that pre-treatment of municipal wastewater is necessary to adjust the pH, the flow or temperature of municipal wastewater when required. Many publications also mention the need for pre-treatment of wastewater to protect the membranes from large particles [3,21,42,43]. For example, Lew et al. conducted anaerobic membrane treatment of pre-settled domestic wastewater to decrease the risk of complications with membrane [4]. Yoo et al. pre-treated the domestic wastewater with 2 mm screening [60]. Of the total of 118 reports, 24 have used a pre-processed feed stream and 29 works treated actual municipal wastewater.

### 5.2. Type of Anaerobic Bioreactor

The CSTR has been widely used, perhaps because of its simple and easy construction for laboratory or pilot use [21,49,50]. A novel AnMBR configuration with a rotating membrane for fouling control is attracting attention nowadays [119]. In this configuration, the rotation provides a shear stress and generates a scouring effect on the membrane surface. In recent work, Ruigomez et al. compared the membrane fouling rates of gas-sparging and rotating modules, and the latter was more successful [120]. They achieved a 0.01 kPa/s fouling rate with the rotational membrane; further reduction of the fouling rate was not possible due to the development of a physically irreversible layer on the membrane surface [120]. However, the rotating AnMBR requires more energy than AnMBR equipped with a gas-sparging system [119].

The anaerobic osmotic membrane bioreactor (AnOMBR) represents an interesting approach to tackling the membrane fouling problem [71,84,121]. Forward osmosis (FO) membranes are characterized by lower fouling rates and better removal efficiency than ultrafiltration (UF) or microfiltration (MF) membranes. As FO membranes are driven by an osmotic gradient, the accumulation of salts remains a large problem [71,84,121]. Wang et al. compared the conventional AnOMBR and a novel MF-coupled AnOMBR system to prevent salt accumulation [84]. As a result, the AnMF–OMBR system was able to operate continuously long term because of the stable salinity level (2.5–4.0 mS/cm) and produced more methane than the conventional system [84].

The UASB reactor has also been used and studied extensively, especially in countries with hot climates, mainly due to low maintenance and operating costs. Moreover, UASB membrane reactors are preferable over continuous flow systems because of they can retain the particulate matter; therefore, the membrane is less affected by pollution [38,44,53,54,56,67,69].

Ozgun et al. studied municipal wastewater treatment with the UASB reactor coupled with a UF system at 15 °C and 25 °C [67]. The high concentrations of COD and microbial products, high turbidity and the small size of particles led to severe membrane fouling at 15 °C compared to 25 °C, while the overall removal performance of the AnMBR was not significantly affected. However, due to the inefficient membrane-fouling control, the MF–UASB is not recommended to use at low temperatures [67].

Other types include bioreactors, such as the gas-lift AnMBR, sponge AnMBR and sponge-granular AnMBR, as well as the AnMBR bioreactors used in research without their type being mentioned in the published articles.

Dolejs et al. investigated the effect of temperature shocks on the gas-lift AnMBR and reported that the gas-lift AnMBR was particularly suited to decentralized wastewater treatment [22]. The system efficiently operated (COD removal >80%) even after short-term decreases to 15 °C.

The introduction of sponge in an AnMBR system may greatly enhance the performance of AnMBR and decrease membrane fouling. Essentially, sponge is a low cost, porous material. It can attach to the biomass and increase the stability of the system under short HRT. Liu et al. added polyester–polyurethane sponges to a conventional submerged AnMBR and reported that membrane longevity was significantly extended, thus improving the filtration performance [86]. The soluble microbial products that largely affect membrane fouling were reduced with the introduction of sponges.

The anaerobic fluidized bed membrane bioreactor (AFMBR) is especially good at dealing with low-strength dilute wastewater [16,93,107]. AFMBR is the hybridization of AnMBR with granular technology [16,93]. Anaerobic sludge granules are strong and have pre-defined shapes and superior settling abilities. They allow the system to maintain a stable biomass under short HRT and other shock conditions [75,78].

Kim et al. used AFMBR with a tubular shape PVDF as a fluidizing agent for the treatment of synthetic wastewater at 36.3 °C and 4–16 h HRT for 240 days in total [107]. The fluidizing agent was effective at decreasing fouling rate due to the scouring effect, thereby maintaining the transmembrane pressure below 0.1 bar and COD removal at more than 90%. The energy consumption was 0.0109 kWh/m^3^, while the energy production from potential methane production was estimated at 0.246 kWh/m^3^, which makes the AFMBR technically feasible [107].

Chen et al. compared the granular AnMBR and sponge-assisted granular AnMBR and reported that the sponge-assisted system yielded sludge granules with better settling and larger particle size [78]. Moreover, the filtration resistance of the sponge–granular AnMBR was 50.7% lower than that of the granular AnMBR, which shows slower membrane fouling development.

### 5.3. Membrane Assembly

Most of the investigations have used membrane-immersed configurations rather than external configurations. This is mainly due to the success of the worldwide application of submerged aerobic MBRs [31].

Indeed, although submerged devices require the bioreactor for membrane cleaning to be shut down, they are still selected by the researchers. Submersible devices have lower energy requirements and less equipment requirements.

Huang et al. used a submerged AnMBR for an investigation of the effect of HRT and SRT on treatment [31]. They reported that the increase in SRT and decrease in HRT led to enhanced microbial growth accelerating the membrane fouling. A side-stream configuration is another interesting strategy to tackle membrane fouling. Vincent et al. reported that the recirculation was able to clean the surface of the membrane but was not capable of removing pollutants inside the pores [88]. However, at this configuration, the mechanical pump is applied to transfer the wastewater which negatively affects the stability of the biomass [28].

Andrade et al. compared internal and external submerged AnMBRs and reported that both configurations were equally efficient at COD removal [10]. However, the external configuration demonstrated superior color removal and less resistance to filtration. Internally submerged AnMBRs experienced membrane fouling as a result of cake formation and subsequent production of exopolymeric substances (EPS).

### 5.4. Type of Membranes

The types of membranes used in the investigations were mainly of three categories: flat sheets, hollow fibers and tubular fibers. Five published studies report the use and study of dynamic membranes. Figure 5 shows the distribution of bibliographic references by type of membrane. The studies seem to outline the use of hollow fiber membranes and subsequently flat plates.

Hollow fiber modules have the largest membrane surface area per unit volume (1200 m^2^/m^3^) among different membranes, which makes them attractive for researchers worldwide, although these membranes are susceptible to blockage by particulate matter due to their narrow diameter (typically in the range of 0.2–2 mm) [122]. The AnMBR with a flat sheet configuration is reported to have a better COD removal rate than hollow fiber or tubular ones; however, a direct comparison of the effect of membrane type on AnMBR performance has yet to be made [36]. Flat sheet membranes can be used in any AnMBR configurations, while hollow fiber and multi-tube membranes are limited to submerged and side-stream configurations, respectively [17]. Tubular modules are almost identical to hollow fiber membranes but have lower surface areas (100 m^2^/m^3^) and tubes of larger diameter (3–25 mm) [122].

### 5.5. Temperature

Regarding the operational parameters of the investigations, Figure 5 shows the number of scientific publications according to the different temperature conditions maintained within the bioreactor for the anaerobic treatment of municipal wastewater.

Three temperature zones were distinguished: 4 °C < 20 °C (cold area—municipal wastewater temperature), 21–40 °C (mesophilic area) and 41–70 °C (thermophilic area). Martinez-Sosa et al. reported that the methane yield was higher in psychrophilic conditions [21]. Under these conditions, methane production is mainly due to hydrogenotrophic methanogenesis [30]. However, microbial activity is decreased at low temperatures and AnMBRs require longer HRTs and SRTs for the removal of pollutants [19]. In addition, the solubility of organic matter also decreases at psychrophilic conditions, which makes membranes susceptible to fouling [63].

It was observed that in more than 60% of the research carried out and included in Table 1, the mesophilic (21–40 °C) is presented as the research conduction temperature, while only a few works operated at thermophilic conditions. Even though mesophilic and thermophilic temperatures are favorable due to the enhanced metabolic activity of anaerobic microorganisms, the additional cost associated with the heating in cold climate countries may reduce interest in this technology [3,41,72].The amount of biogas generated from the treatment of low-strength municipal wastewater is not enough to cover the heating energy consumption [2,36]. As an alternative, the heat of the discharge wastewater can be extracted using heat-pump technology, which can significantly reduce energy consumption [2]. Currently, AnMBR is highly suitable for tropical countries and the technology should be optimized at ambient temperatures to be economically viable [2].

### 5.6. COD Removal

As revealed by most of the research under consideration, AnMBR technology is extremely efficient in removing organic load from the feed stream. The filtrate showed satisfactory COD removal, greater than 90% and in some cases 98–99%, indicating that AnMBR technology is an extremely competitive municipal wastewater treatment technique (Figure 5).

COD removal by AnMBR happens primarily by biological (in the bioreactor) and to a lesser extent by physical (membrane filtration and adsorption) mechanisms [36,63,123]. It is also possible that some bacteria are located on the membrane surface forming the gel or cake layer [21,29,67,73,83]. This layer, which is also termed a dynamic membrane, can further enhance the COD removal efficiency of the process by the rejection of soluble low-molecular weight compounds [47]. The presence of soluble COD leads to a higher membrane fouling rate due to a decrease of the membrane flux [21,26]. Interestingly, the membranes with high fouling rates also have high concentrations of EPS, which can increase the adsorption rate of soluble organic compounds [36]. The presence of metal ions adversely affects COD removal due to their toxicity [100,124]. On the contrary, the increase of conductivity by the addition of salts slightly increases the removal of organics [62].

Ozgun et al. reported that COD removal depends on the temperature of the bioreactor [67]. At 25 °C the removal rate of COD was 92%, while it decreased to 90% when the temperature was reduced to 15 °C. The result is also confirmed by research by Ho and Sung who also observed that the COD removal rate was 85% at 15 °C and 95% at 25 °C [43].

Pretel et al. investigated the effect of temperature, SRT and HRT functional parameters on COD removal [6]. They report that to achieve the same rate of COD removal when the temperature drops the hydraulic retention time and sludge retention time should be increased. Specifically, when T = 30 °C then HRT = 7 h and SRT = 12 days, whereas when T = 15 °C then HRT = 14 h and SRT = 35 days are required to achieve the same rate of COD removal.

Khan et al. conducted experiments in a pH range of 5–12 at constant HRT and reported that the maximum COD removal was 79.8% when pH was kept at 7 [96].

Smith et al. showed that, when treated under the same conditions and in the same AnMBR, the COD removal rate in samples of real municipal wastewater and simulated municipal wastewater was 69% and 92%, respectively [36]. The authors linked this significant difference in COD removal with the lower strength of real wastewater than of the synthetic one (259 mg/L versus 440 mg/L).

Chen et al. demonstrated that COD removal in a synthetic wastewater sample and a synthetic wastewater sample with ZnO nanoparticles added decreased from 96.4% in the first case to 81.5% in the second case [100]. Zn^2+^ ions had a toxic effect on anaerobic microorganisms, thereby reducing their efficiency. A significant decrease in biogas production was also observed.

The same results were obtained by Do and Stuckey who stated that the removal rate of COD decreased from 89% to 78% when the same treatment was performed on synthetic wastewater containing a significant amount of ciprofloxacin (4.7 mg/L) antibiotic [98]. The antibiotic restricted the growth of anaerobic microbes.

### 5.7. COD Removal–Addition of Activated Carbon

The addition of activated carbon to the AnMBR systems was investigated, both to remove organic load and micropollutants. Activated carbon has a high absorption capacity for macromolecules and provides a surface for biomass adhesion. Lim et al. reported that the addition of activated carbon improved the rate of removal of organic material [93]. Specifically, at T = 25 °C, the COD removal rate was 96.2% due to the addition of activated carbon. In contrast, Xiao et al. showed that at temperature T = 35 °C, the removal rate of COD = 93.8% was not affected by the addition of activated carbon [33]. However, the removal rate of five studied pharmaceutical substances (trimethoprim, sulfamethoxazole, carbamazepine, diclofenac and triclosan) significantly increased.

### 5.8. Production of Methane/Biogas

**Figure 6:**
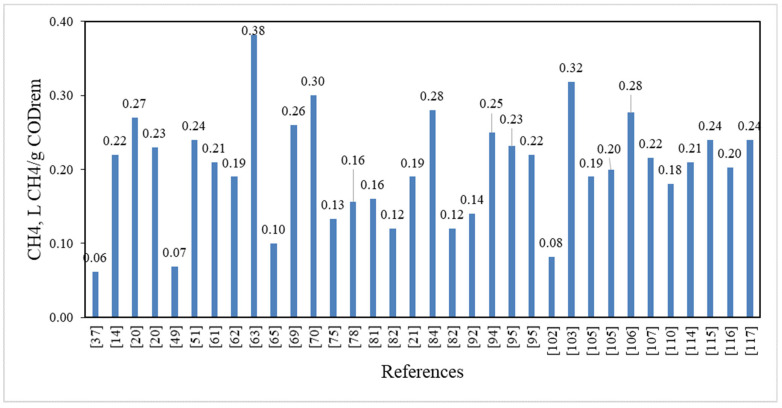
Methane production during anaerobic biodegradation of municipal wastewater in an AnMBR system.

Concerning the performance of the AnMBR system in biogas production, Figure 6 presents the results of the research reported in Table 1 of this paper. To the best of our knowledge, the highest methane production was achieved by Wei et al. with a value of 0.382 LCH_4_/gCOD at 35 °C [64].

Production of methane from “weak” municipal wastewater is one of the major advantages of AnMBR technology. It has been shown that up to 98% of COD can be converted to biogas under specific conditions of anaerobic treatment. The most important and determining parameters are organic load [56,85] and temperature [21]. At ambient temperature, a significant amount of methane is dissolved and lost in the filtrate; approximately 30–40% of the amount produced is lost in the permeate. At low temperatures, the solubility of CO_2_ is greater than that of CH_4_. At 35 °C, biogas production and utilization are improved. Gimenez et al. [54] reported that CH_4_ production at 20 °C was 53.6%, while at 33 °C it increased to 57.4%. Smith et al. [36] also claimed that at 15 °C only 40–50% of the methane produced was recovered.

Temperature plays an important role in biogas production [3,54,104]. It has been reported that the activity of methanogenic archaea is affected at psychrophilic temperatures, decreasing the amount of the biogas produced [54]. Moreover, low temperatures change the composition of the biogas, shrinking the proportion of methane. This could be explained by the increase of solubility of methane in water at lower temperatures. Thus, more methane escapes the reactor with the effluent decreasing the proportion of methane in biogas [3,21,22,50,125]. In AnMBR, the removal of soluble COD is mainly due to the microorganisms, and biofilm can produce additional methane. However, this, coupled with membrane pressure gradients, can also increase the solubility of methane in water, leading to the loss of produced methane [51]. Similarly, the larger size of sludge particles also leads to poor methane production through mass transfer limitations [56].

It was reported that a lower oxidation reduction potential (ORP) can intensify methanogenesis [104]. It is possible that the biochar used by Chen et al. provided optimal ORP conditions for microorganisms to convert propionic acid to methane. In another work, Chen et al. investigated the effect of granular activated carbon (GAC) on methane production [115]. Specifically, Chen et al. found that *Methanosaeta*, which is responsible for methane generation, was more active in GAC-sludge samples even under low temperatures (15–5 °C).

Regarding organic loading, there seems to be a linear correlation with biogas production. Hu et al. found that for OLR = 0.88 kgCO)/m^3^d, 1.55 kgCOD/m^3^d and 3.01 kgCOD/m^3^d, the biogas produced is 0.3 L/d, 0.41 L/d and 1.56 L/d, respectively [85]. Aslam et al. demonstrated that when the organic load is 0.46 kgCOD/m^3^d, the highest rate of COD removal and biogas production is influenced by the feed stream flow density and is achieved when the flux density is 6 L/m^2^*h. The biogas production was then 0.44 L/gCOD [94].

**Figure 7:**
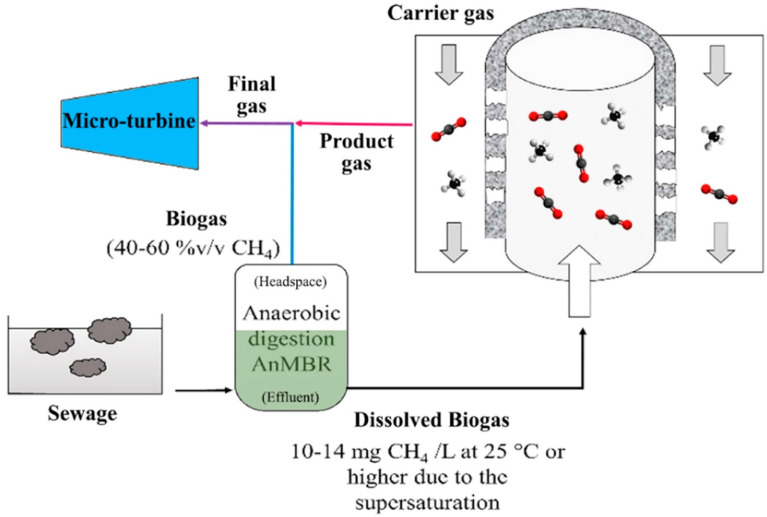
Method for recovering dissolved methane from AnMBR systems outflow. Reprinted from ref. [89], copyright (2018), with permission from Elsevier.

Rongwong et al. found that methane production increases linearly with COD increase in the reactor (Figure 7) [89,90]. Specifically, for COD 350, 500, 650, 750 mg/L, methane production is 110, 157, 204 and 236 L/m^3^, respectively. The percentage of methane lost in the filtrate is 30, 21, 16 and 14%, respectively. By a suitable method, this percentage of methane can be recovered up to 85.37%.

Generally, optimum biogas production is achieved at high HRT and SRT values [126]. Ho and Sung investigated the effect of HRT change on biogas production by maintaining a high SRT of 90–360 days and found that reducing HRT from 12 to 6 h resulted in a reduction of recovered methane from 48% to 35% [15].

Noyola et al. stated that the presence of sulfur compounds in wastewater has the effect of promoting the growth of sulfur-reducing bacteria at the expense of methanogens [127]. The conversion of SO_4_^2−^ to S^2−^ competes with the production of CH_4_ and produces a toxic and corrosive gas, H_2_S. Vincent et al. reported that wastewater rich in sulfates should be avoided in AnMBR systems [88].

Chen et al. demonstrated that, when treated under the same conditions and in the same AnMBR, a significant decrease in biogas production was observed in a synthetic wastewater sample with ZnO NPs added compared to an unadulterated synthetic wastewater sample [100]. From 0.36–0.42, production decreased to 0.11 L/gCOD and became zero in the anaerobic process. Zn^2+^ ions had a toxic effect on anaerobic microorganisms, thereby reducing their efficiency.

In addition, AnMBR technology allows the recovery of intermediate products, such as H_2_, which can be used as fuel. Ferreira et al. claimed that from synthetic wastewater 5000 mg/L COD, in mesophilic conditions and HRT = 1 h, 344.9 ± 74 mlH_2_/hL can be produced [128].

To stabilize the organic constituents in the anaerobic bioreactor, no significant amount of energy is required, as is the case with the conventional active sludge method, in which high amounts of energy are wasted to aerate the wastewater and provide aerobic conditions for the metabolism of the microorganisms. The energy consumption for membrane filtration can be offset by utilizing the methane produced [72].

It is clear that research on AnMBR systems needs to be expanded to better identify the conditions under which anaerobic wastewater treatment with a membrane treatment system is practical and economically feasible.

### 5.9. Micropollutant Removal

The removal of micropollutants in wastewater treatment can be attributed to adsorption and biotransformation. In AnMBR systems, removal via biotransformation prevails over sludge absorption. The high residence time of the sludge obtained with the membranes increases the efficiency of the removal of micropollutants, such as pharmaceutical substances, as it increases the exposure time to the slowly growing anaerobic microbial populations. The use of highly selective membranes and the addition of materials with adsorbent properties (sponge, activated carbon) significantly increases the removal rate of the micropollutants.

Zhu et al. studied the performance of AnMBR treating 2-chlorophenol wastewater [109]. They reported that the presence of toxic 2-chlorophenol stimulated the increase in EPS proteins, which further increased the membrane fouling rate. Do and Stuckey investigated the removal of ciprofloxacin in a batch AnMBR and achieved 50–76% removal of the target pollutant [98]. The adsorption kinetics studies revealed that adsorption of ciprofloxacin onto anaerobic sludge happens rapidly due to the availability of adsorption sites (75% of adsorption in 10 min). However, after that adsorption slows down, reaching the equilibrium in 90 min. At most, adsorption was responsible for 26% removal of ciprofloxacin, biological degradation being the major mechanism for ciprofloxacin removal. Similar findings were reported by Liu et al. [106]. In their work on the removal of trace organic contaminants (TrOCs), only amitriptyline, 4-tert-octyphenol and triclosan out of 15 TrOCs were adsorbed onto sludge in a range of 2–3% during AnMBR treatment. Monsalvo et al. studied the application of AnMBRs for the removal of 38 TrOCs and reported more than 90% removal of nine compounds and less than 50% removal of 23 of compounds [61]. The TrOCs were removed mainly by biodegradation and partially by adsorption.

**Figure 8:**
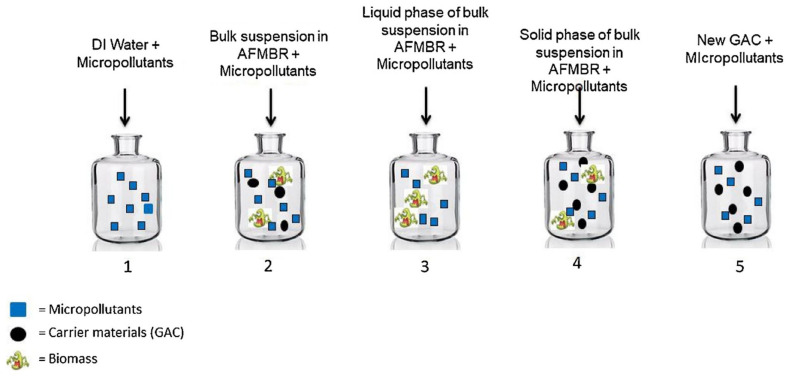
Schematic illustration of experiments by Lim et al. [93] to investigate the mechanism of pharmaceutical substances removal from municipal wastewater. Reprinted from ref. [93], copyright (2019), with permission from Elsevier.

Wei et al. presented 87% retention of organic micropollutants when 100 mg/L of activated carbon was added to the bioreactor [74]. Lim et al. illustrated complete removal of three pharmaceutical substances (diclofenac, ibuprofen and sulfamethoxazole) from synthetic domestic wastewater treated with an AnMBR [93]. Particularly important is the effect of adding activated carbon to the bioreactor. Lim et al. found that the pharmaceutical substances diclofenac, ibuprofen and sulfamethoxazole were 100% removed when activated carbon was added [93]. Specifically, they performed five experiments on an AnMBR system (Figure 8).

**Figure 9:**
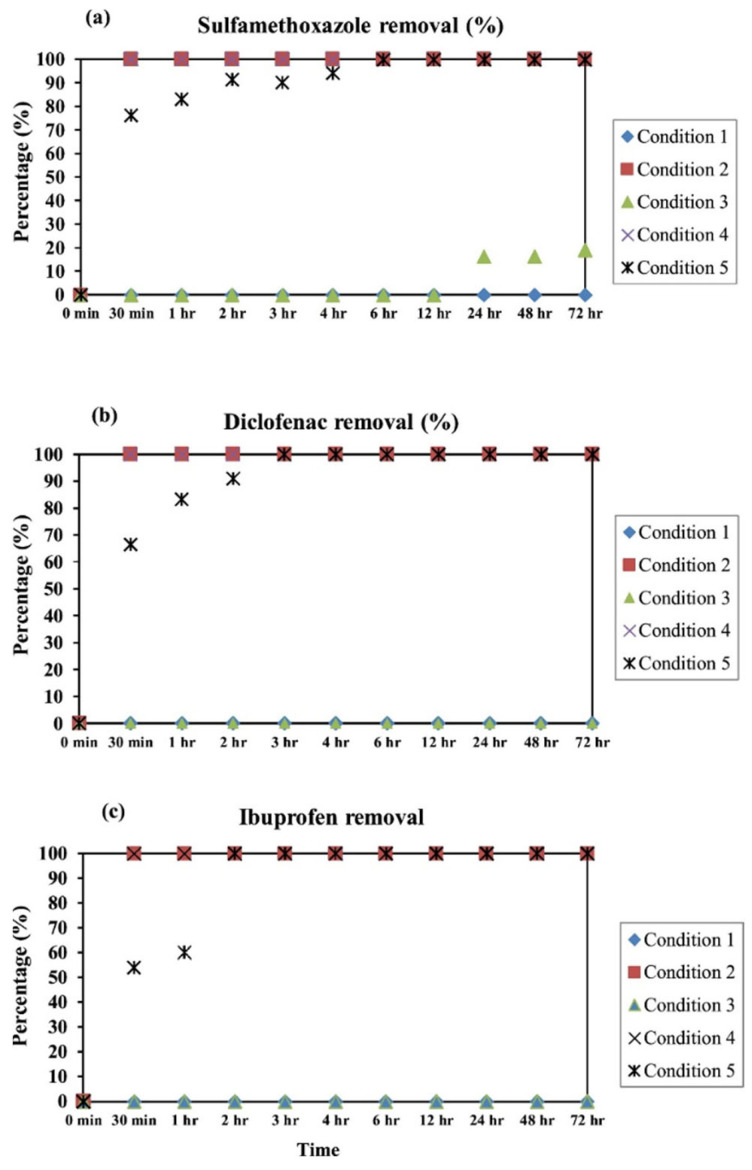
Removal effects of the pharmaceuticals (**a**) sulfamethoxazole, (**b**) diclofenac and (**c**) ibuprofen in each case. Reprinted from ref. [93], copyright (2019), with permission from Elsevier.

The results of the research, i.e., the rate of pharmaceutical substance removal, are plotted in Figure 9.

In the second case (condition 2), which used feed stream micropollutants + suspension of activated carbon granules + biomass, 100% pharmaceutical substance removal was observed. The results of the research are in agreement with other bibliographical references [33,34,61].

**Figure 10:**
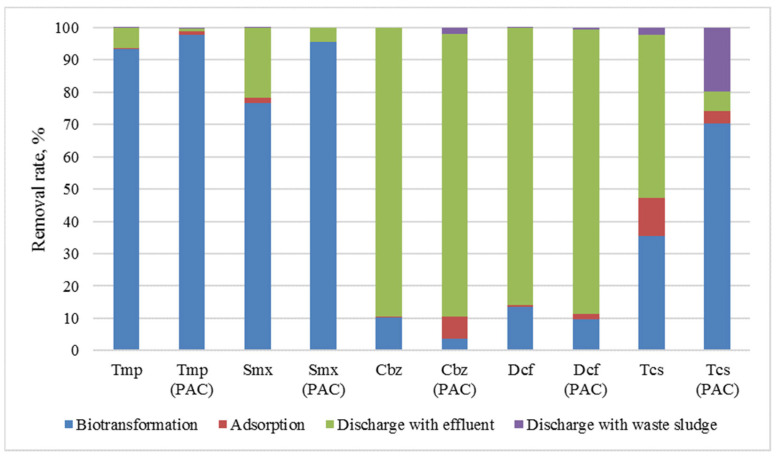
Methods and percentages of pharmaceutical substance removal in an AnMBR system before and after the addition of PAC. Adapted from ref. [33], copyright (2017), with permission from Elsevier.

Xiao et al. report that the removal of five pharmaceutical substances, namely, carbamazepine (CBZ), diclofenac (DCF), triclosan (TCF), sulfamethoxazole (SMX) and trimethoprim (TMP), was significantly improved when powdered activated carbon (PAC) was added in the bioreactor, at least in the first five days of addition (Figure 10) [33].

AnMBR systems produce nutrient-rich effluents (nitrogen and phosphorus) that can be utilized for irrigation [21,92]. However, there is still concern about the reuse of effluent in irrigation, which is related to water quality and in particular the presence of pathogens, viruses and other substances, such as heavy metals and emerging pollutants, e.g., pharmaceutical substances. Further research is needed to assess and study the potential risks to human health and the ecosystem.

## 6. Conclusions and Future Research

The literature review of the present work has shown that AnMBR systems can effectively treat municipal wastewater and produce a high-quality effluent. It is an environmentally friendly green technology with the possibility of utilizing the produced biogas for electricity and reusing treated water for irrigation.

The use of an external AnMBR may reduce the possibility of membrane fouling; however, it increases the overall cost of the AnMBR system. Therefore, AnMBRs with submerged membranes are prevalent. The efficiency of an AnMBR largely depends on process parameters, such as temperature, pH, alkalinity, SRT, HRT and the concentration of pollutants. Anaerobic digestion favors mesophilic and thermophilic temperature zones, while efficiency significantly decreases at lower temperatures. AnMBR systems allow the production of methane even from low-strength wastewater, such as municipal wastewater, and at ambient temperatures. However, research is still needed to ensure the recovery of methane whose solubility increases at low temperatures and is lost to effluent.

Two-stage configurations of AnMBRs have been developed to ensure optimal methane production conditions for both acidogenesis (pH 5.5–6.5) and methanogenesis (pH 7). Moreover, the concentration of VFAs should be maintained at a specific range to prevent a decrease in pH, which results in an inhibition of methanogenesis. Alkalinity plays a crucial role in the pH stability of the system, where the VFA/TA ratio should be kept at a value less than 0.4. Fluctuations in the influent OLR are reported to not have an effect on the efficiency of AnMBRs, although a high OLR may result in the accumulation of VFAs. AnMBRs produce less sludge than the activated sludge process due to their high SRT, which eases sludge disposal management. However, as with aerobic MBR technology, the most significant disadvantage of AnMBR systems is membrane fouling. The blockage, which increases the hydraulic residence time, has limited the widespread application of membrane technology.

Membranes coupled with biological treatment can to a certain extent remove micropollutants, such as pharmaceutical substances and substances coming from personal care products, but additional treatment is often required to maximize the removal efficiency. In this case, the economic viability of the method must be considered.

While AnMBR technology has a significant number of advantages over conventional systems, many problems still need to be optimized. AnMBR systems appear, at least on a pilot laboratory level, to have a competitive advantage over conventional active sludge treatment in municipal wastewater treatment. However, in the years to come, this technology does not seem likely to prevail. Full-scale research is still needed to produce a well-studied, mature technology.

Stricter limits on the disposal and reuse of treated municipal wastewater and the ever-evolving analytical methods for identifying even trace chemicals in environmental samples may give impetus to upgrading existing WWTPs or replacing them with innovative treatment technologies, specifically in areas where a high degree of protection must be achieved. Of course, upgrading or replacing existing municipal wastewater treatment systems largely depends on the financial burdens and potential impacts on the maintenance of a WWTP.

## Data Availability

Data available on request.
